# The POSH-Ex Study: Study Protocol for a Randomized Controlled Trial of a 12-Week Online Physiotherapy Intervention for Improving Postpartum Sexual Health

**DOI:** 10.3390/healthcare14193277

**Published:** 2026-10-02

**Authors:** Nikolina Zaplatić Degač, Ivana Živoder, Špela Bogataj

**Affiliations:** 1 Faculty of Health Sciences, University of Novo mesto, Na Loko 2, 8000 Novo Mesto, Slovenia; 2 Department of Physiotherapy, University North, Trg dr. Žarka Dolinara 1, 48000 Koprivnica, Croatia; izivoder@unin.hr; 3 Department of Nephrology, University Medical Centre Ljubljana, Zaloška Cesta 7, 1000 Ljubljana, Slovenia; spela.bogataj@kclj.si; 4 Faculty of Sport, University of Ljubljana, Gortanova 22, 1000 Ljubljana, Slovenia

**Keywords:** postpartum period, sexual health, female sexual function, therapeutic exercise, telerehabilitation, physiotherapy

## Abstract

**Highlights:**

**What are the main findings?**

- The POSH-Ex study introduces a structured 12-week online physiotherapy programme integrating pelvic floor muscle training, lumbopelvic stabilisation, functional strengthening, breathing, and movement control to support postpartum sexual health.
- The protocol applies a four-phase progressive exercise model and evaluates sexual function alongside physical activity, fatigue, and depressive symptoms in postpartum women.

**What are the implications of the main findings?**

- If effective, POSH-Ex may provide a structured and reproducible framework for integrating pelvic floor rehabilitation with whole-body functional exercise in postpartum physiotherapy.
- The findings of the trial may inform future physiotherapy practice and the development of structured, remotely delivered exercise programmes addressing postpartum sexual health and related aspects of recovery.

**Abstract:**

Background/Objectives: Postpartum sexual dysfunction is common and multifactorial, yet structured physiotherapy interventions specifically targeting sexual function after childbirth remain insufficiently investigated. This study aims to evaluate the effectiveness of Postpartum Sexual Health Exercise (POSH-Ex), a standardized, multimodal, physiotherapist-led online therapeutic exercise programme designed to improve postpartum sexual function. We hypothesise that participants allocated to POSH-Ex will have a higher mean FSFI total score at 12 weeks than participants receiving usual postpartum care, after adjustment for baseline FSFI score. Methods: This randomized controlled trial will include 84 women recruited at 10–12 weeks postpartum and randomly allocated (1:1) to the POSH-Ex intervention or control group. The intervention will consist of a 12-week progressive programme delivered twice weekly in 60-min sessions, integrating pelvic floor muscle training, breathing and relaxation exercises, trunk stabilization, functional strengthening, flexibility, and movement re-education, while the control group will receive usual postpartum care and continue their usual activities without a study-specific exercise intervention. Outcomes will be assessed at baseline and after 12 weeks. Sexual function, assessed using the Female Sexual Function Index (FSFI), is the primary outcome; physical activity (IPAQ-SF), fatigue (FAS), and depressive symptoms (EPDS) are secondary outcomes. The primary analysis will compare FSFI scores at 12 weeks between groups using analysis of covariance (ANCOVA) adjusted for baseline FSFI. The trial was prospectively registered at ClinicalTrials.gov (NCT07747285). Conclusions: The POSH-Ex trial is designed to determine whether a structured, multimodal, physiotherapist-led online therapeutic exercise programme improves postpartum sexual function compared with usual postpartum care. If effective, POSH-Ex may inform the development of structured physiotherapy approaches that recognise sexual health as an integral component of comprehensive postpartum care.

## 1. Introduction

The postpartum period, often referred to as the “fourth trimester”, is characterised by substantial physiological, psychological, and social changes as women recover from pregnancy and childbirth while adapting to motherhood. The American College of Obstetricians and Gynecologists recommends that postpartum care be considered an ongoing, woman-centred process rather than a single clinical encounter, with services and support tailored to each woman’s individual needs [1]. Nevertheless, important gaps in comprehensive and coordinated postpartum care persist, including fragmentation of services, discontinuity of care, and unmet needs for information and support, which may leave important maternal health concerns insufficiently recognised and addressed [2,3].

Fatigue, depressive symptoms, and sexual concerns are common during postpartum recovery and may coexist as interconnected dimensions of maternal health. Postpartum fatigue affects more than 60% of women and may persist for several months, adversely affecting daily functioning, caregiving capacity, and psychological well-being [4]. Persistent fatigue is also associated with postpartum depressive symptoms [5], while postpartum depression affects approximately 10–20% of women and may adversely affect maternal functioning, partner relationships, and child development [6]. Sexual health is similarly multidimensional and is shaped by interacting biological, psychological, relational, and social factors [7]. Sexual dysfunction is frequently reported during the first six months postpartum, with estimates ranging from 40% to 80%, and may include reduced sexual desire, dyspareunia, and diminished sexual satisfaction [8,9]. Despite its relevance to women’s quality of life and postpartum well-being, sexual concerns remain insufficiently addressed in postpartum care, and women may be reluctant to initiate discussions about these concerns with healthcare professionals [10].

Physiotherapist-led therapeutic exercise represents a promising non-pharmacological approach to postpartum recovery. Unlike general physical activity, therapeutic exercise can be individually prescribed, progressively adapted, and professionally supervised according to a woman’s functional status, movement quality, symptom response, and recovery needs. Evidence from systematic reviews and meta-analyses indicates that postpartum exercise may reduce depressive and anxiety symptoms [11,12], while structured pelvic floor muscle exercise may also contribute to improvements in sexual function [13,14]. Nevertheless, maintaining regular physical activity during the postpartum period can be challenging, with fatigue, limited time, childcare and maternal responsibilities, and reduced motivation commonly reported as barriers to participation [15,16].

The potential benefits of therapeutic exercise can be understood within a biopsychosocial framework. Physiologically, the pelvic floor muscles contribute to postural and respiratory functions and are involved in lumbopelvic control [17,18], while pelvic floor muscle function and training are also relevant to continence and postpartum sexual function [14,18]. From a psychological and functional perspective, exercise may support exercise-related self-efficacy and contribute to reductions in stress and fatigue during the postpartum period [19,20,21,22]. Sexual function during this period is also associated with a range of obstetric, psychological, and relational factors, including breastfeeding, perineal trauma, body image, and partner support [23,24,25,26], further emphasising the multifactorial nature of postpartum recovery.

Despite growing evidence supporting postpartum exercise, important knowledge gaps remain. Previous studies have reported favourable effects of exercise on individual postpartum outcomes including fatigue, depressive symptoms, and perceived stress, while higher levels of physical activity have been associated with lower perceived fatigue [19,27,28,29]. However, existing postpartum exercise interventions have often focused on specific components of recovery, such as pelvic floor muscle training, management of pelvic floor dysfunction, depressive symptoms, fatigue, or general physical recovery [13,14,27,28,29]. Although pelvic floor muscle training has been investigated in relation to postpartum sexual function, the available evidence remains limited and heterogeneous [13,14]. There therefore remains a need for well-defined and reproducible postpartum exercise programmes that integrate multiple therapeutic exercise components within a structured and progressive intervention, particularly given the heterogeneity of existing exercise approaches and the limited evidence regarding their effects on postpartum sexual function [14,30].

To address these knowledge gaps, we developed the Postpartum Sexual Health Exercise (POSH-Ex) intervention, a standardised, multimodal, physiotherapist-led therapeutic exercise programme designed for women in the postpartum period. POSH-Ex integrates pelvic floor muscle activation and relaxation, breathing coordination, trunk stabilisation, functional strengthening, flexibility, and movement re-education within a structured 12-week programme delivered through synchronous online physiotherapist supervision. We hypothesise that participants allocated to POSH-Ex will have a higher mean FSFI total score at 12 weeks than participants receiving usual postpartum care, after adjustment for baseline FSFI score.

## 2. Materials and Methods

### 2.1. Study Design

This study will be conducted as a prospective, two-arm, parallel-group randomised controlled trial with a 1:1 allocation ratio. Participants will be randomly assigned to either the intervention group, which will participate in the 12-week Postpartum Sexual Health Exercise (POSH-Ex) programme, or the control group, which will not receive the POSH-Ex intervention and will continue their usual postpartum care and daily activities. Outcomes will be assessed at baseline (T0) and immediately following the 12-week intervention period (T1). The protocol will be reported in accordance with the Standard Protocol Items: Recommendations for Interventional Trials (SPIRIT) guidelines, while the subsequent reporting of trial results will follow the Consolidated Standards of Reporting Trials (CONSORT) recommendations.

### 2.2. Study Setting

The study will be conducted in the Republic of Croatia using a remote, home-based intervention model. The POSH-Ex intervention will be delivered through synchronous videoconferencing using Google Meet, allowing participants to take part from their home environment while receiving real-time supervision and feedback from a physiotherapist. This delivery model is intended to reduce geographical, travel, and time-related barriers to participation during the postpartum period while maintaining direct professional supervision throughout the intervention.

### 2.3. Ethics and Trial Registration

The study will be conducted in accordance with the ethical principles of the Declaration of Helsinki. Ethical approval has been obtained from the Ethics Committee of the Faculty of Health Sciences, University of Novo mesto (approval No. UNM 118/2026). All participants will receive written and verbal information about the study and will provide written informed consent before enrolment. Participation will be entirely voluntary, and participants will have the right to withdraw from the study at any time without providing a reason and without any consequences. Participant confidentiality will be protected through pseudonymisation, and all personal and research data will be handled in accordance with applicable data protection requirements, including the General Data Protection Regulation (GDPR).

The trial was prospectively registered on 4 August 2026 at ClinicalTrials.gov prior to enrolment of the first participant (ClinicalTrials.gov identifier: NCT07747285). Any additional institutional approvals required at participating recruitment sites will be obtained before recruitment is initiated at the respective site.

### 2.4. Participants and Eligibility Criteria

Women in the early postpartum period will be eligible to participate in the study if they meet all predefined inclusion criteria. Eligibility will be assessed prior to enrolment by the investigator, who is a physiotherapist, based on the available medical documentation and the predefined eligibility criteria, to ensure participant safety. Safety for participation in moderate-intensity therapeutic exercise will be evaluated in accordance with recommendations of the American College of Obstetricians and Gynecologists (ACOG), which support gradual resumption of postpartum exercise when medically safe, taking into account the mode of delivery and the presence of medical or surgical complications [31].

The inclusion criteria will be: (1) women aged 18–45 years; (2) 10–12 weeks postpartum at baseline assessment; (3) live birth; (4) ability to understand and communicate in Croatian; (5) ability to independently complete the study questionnaires; (6) access to an internet-enabled device with a camera and stable internet connection required for participation in the online intervention; and (7) provision of written informed consent.

The exclusion criteria will be: (1) any documented medical contraindication to participation in moderate-intensity therapeutic exercise; (2) unresolved acute postpartum complications including ongoing significant bleeding, infection, impaired wound healing, symptomatic cardiovascular or thromboembolic conditions, or other clinically significant postpartum conditions documented as requiring restriction of physical exercise; (3) a documented medical, neurological, musculoskeletal, or psychiatric condition that would preclude safe performance of the prescribed exercises or reliable completion of the study assessments; (4) current participation, at the time of enrolment, in another structured exercise, rehabilitation, or physiotherapy programme that could reasonably influence the study outcomes; (5) inability to participate in the online supervised sessions; and (6) inability to comply with the study procedures.

### 2.5. Recruitment and Informed Consent

Potential participants will be identified and approached by community health nurses from participating primary healthcare centres in Croatia during routine postpartum care. The nurses will provide eligible women with brief information about the study and, with their permission, facilitate contact with the research team. Women who express an interest in participating will receive detailed written and verbal information regarding the study objectives, procedures, intervention, potential benefits and risks, confidentiality, and their right to withdraw from the study at any time without consequences for their healthcare.

Women who meet the eligibility criteria and agree to participate will provide written informed consent before any study-specific procedures or baseline assessments are performed. Participation will be entirely voluntary, and refusal to participate or subsequent withdrawal from the study will not affect the healthcare received by the participant.

### 2.6. Randomisation and Allocation Concealment

The trial will be conducted in several consecutive recruitment cycles; therefore, participants will not all receive the intervention at the same time. A recruitment cycle is defined as a predefined enrolment period during which eligible women provide informed consent and complete the baseline assessment. Following completion of the baseline assessment (T0) of all participants enrolled in a given cycle, including review of the baseline EPDS responses as described in Section 2.9.2, these participants will be randomly allocated to either the POSH-Ex intervention group or the control group in a 1:1 ratio using block randomisation, with each recruitment cycle forming a separate randomisation block. Participants allocated to the intervention group within a cycle will then start and complete the 12-week POSH-Ex programme together, while participants allocated to the control group will start the 12-week study period on the same date. Subsequent recruitment cycles, each with a new randomisation, will be conducted at later dates, and the intervention will be repeated in each cycle until the target sample of 84 randomised participants has been reached (Section 2.12). This cohort-based structure is required because the synchronous POSH-Ex sessions are delivered on a fixed weekly schedule by the same physiotherapist. Randomisation will be performed separately for each recruitment cycle; recruitment cycle is therefore the stratification factor, which ensures balanced allocation between the two study groups within each cycle and across the calendar periods in which participants are enrolled.

The allocation sequence will be computer-generated by an independent person who will not be involved in participant recruitment, baseline assessment, intervention delivery, or data analysis. For each recruitment cycle, once the baseline assessment of all participants in that cycle has been completed, the principal investigator will send only the list of participant identification codes for that cycle to the independent person, who will allocate these participants to the two groups in a 1:1 ratio using Research Randomizer (www.randomizer.org) [32]. The participants in the list will be numbered consecutively: one set of unique random numbers covering all positions in the list (i.e., a random permutation) will be generated, and the participants corresponding to the first half of the permuted sequence will be allocated to the POSH-Ex group and the remaining participants to the control group; if a cycle includes an odd number of participants, the participant in the middle position of the permuted sequence will be allocated to either group by an additional random draw. The output of each randomisation will be saved and archived by the independent person. Each cycle therefore constitutes one randomisation block, the size of which equals the number of participants enrolled in the cycle and consequently varies between cycles. No further stratification by participant characteristics will be applied, because the planned sample size does not allow several stratification factors to be used without the risk of sparsely populated strata; instead, prognostically relevant baseline characteristics will be summarised by group and considered in a prespecified covariate-adjusted sensitivity analysis (Section 2.13). To ensure allocation concealment, the randomisation sequence will remain inaccessible to the research team responsible for participant recruitment and baseline assessment. Because all participants within a cycle will be randomised simultaneously, and only after their enrolment and baseline assessment have been completed, group allocation cannot be known or predicted during recruitment or baseline assessment. Following allocation, participants will be informed of their group assignment and provided with the corresponding study instructions.

### 2.7. Blinding

Due to the nature of the intervention, blinding of participants and the physiotherapist delivering the POSH-Ex programme will not be possible. The primary and secondary outcomes will be assessed using self-administered questionnaires completed by participants at baseline (T0) and immediately following the 12-week intervention period (T1); therefore, no separate outcome assessor will be involved in outcome measurement.

To minimise potential bias, group allocation will remain concealed until completion of the baseline assessment. All outcome data will be coded using unique participant identification numbers prior to analysis. Statistical analyses will be performed by an independent statistician who will not be involved in participant recruitment, randomisation, intervention delivery, or data collection. The statistician will receive a coded dataset in which the intervention and control groups are identified using neutral group labels, thereby remaining blinded to group identity during the primary statistical analyses. Group identities will be revealed only after completion of the prespecified primary analyses.

### 2.8. Intervention

#### 2.8.1. POSH-Ex Intervention

Participants allocated to the intervention group will receive the Postpartum Sexual Health Exercise (POSH-Ex) programme in addition to usual postpartum care. POSH-Ex is a standardised, multimodal, 12-week, physiotherapist-led therapeutic exercise intervention developed for women in the postpartum period. The programme was developed on the basis of current evidence and contemporary principles of postpartum rehabilitation, with particular emphasis on pelvic floor muscle function, lumbopelvic stability, regulation of intra-abdominal pressure, functional integration of the trunk and lower extremities, and progressive return to physical activity [30]. It integrates diaphragmatic breathing, pelvic floor muscle relaxation and activation, neuromuscular control, trunk stabilisation, functional strengthening, flexibility, movement re-education, and progressively more complex functional movement patterns. These components are intended to address interconnected neuromuscular and functional mechanisms relevant to postpartum recovery and sexual health [33].

The POSH-Ex intervention will be delivered over 12 consecutive weeks, with two 60-min exercise sessions scheduled per week. Sessions will be conducted synchronously via Google Meet and supervised by the same physiotherapist throughout the intervention period to ensure consistency in exercise instruction, progression, feedback, and monitoring. Participants will join the online sessions using the pseudonym assigned at enrolment and will be required to keep their cameras switched on during supervised sessions. Real-time visual supervision will enable the physiotherapist to monitor exercise technique and movement quality, identify and correct inappropriate movement patterns, assess symptom responses, and modify exercises when necessary. No video or audio recordings of the sessions will be made.

Given the specific needs of women in the early postpartum period, participants will be permitted to attend synchronous sessions with their infants present. Brief interruptions related to infant feeding, soothing, or other immediate caregiving needs will be permitted during the live session, after which the participant may resume the exercise programme when able to do so safely. Such brief interruptions will not be considered protocol deviations.

At the beginning of each week, participants will receive standardised exercise materials by email containing illustrated and written instructions for the exercises prescribed for that week, including exercise dosage and key technical guidance. Separate pre-recorded video demonstrations will not be provided; exercise demonstration, instruction, and correction will be delivered during the synchronous physiotherapist-supervised sessions. If a participant is unable to attend one of the two scheduled supervised sessions, she will be instructed to complete the corresponding session independently during the same week using the provided materials and to record its completion in an exercise diary. Participants will be required to attend at least one physiotherapist-supervised session per week to maintain regular professional supervision. Attendance at supervised sessions and completion of independently performed replacement sessions will be documented separately throughout the intervention.

The POSH-Ex intervention is described in accordance with the Template for Intervention Description and Replication (TIDieR) and the Consensus on Exercise Reporting Template (CERT) to support transparent reporting and reproducibility [34,35].

#### 2.8.2. Programme Structure and Progression

The POSH-Ex programme comprises a standardised core exercise protocol, while individualisation is limited to prespecified progression, regression, and exercise-modification criteria based on movement quality, functional capacity, and symptom response. Thus, all participants will follow the same overall programme structure and therapeutic principles, with adaptations made within the predefined intervention framework.

The POSH-Ex programme will follow a four-phase progressive structure, with each phase lasting three weeks (Table 1). The intervention will progress from neuromuscular regulation, pelvic floor muscle awareness, breathing coordination, and basic lumbopelvic control in Phase I, through functional endurance and integrated lumbopelvic stability in Phase II, to functional strengthening and dynamic lumbopelvic stability in Phase III, culminating in advanced functional integration and movement control in Phase IV.

Progression will be achieved through gradual increases in exercise duration, time under tension, motor complexity, functional loading, coordination, balance demands, and movement integration, depending on the objectives of each phase. External resistance will be introduced progressively where appropriate. Throughout the programme, progression will be individualised according to movement quality, symptom response, and the participant’s ability to maintain coordinated breathing, appropriate pelvic floor muscle activation and relaxation, regulation of intra-abdominal pressure, and lumbopelvic control.

Progression between phases will be based on predefined functional and safety criteria rather than solely on completion of the scheduled three-week period. Participants who do not meet the criteria for progression will continue with the appropriate exercises from the preceding phase or receive modified exercise variants until the required criteria are achieved. Exercise intensity will progress across the four intervention phases, from RPE 4–5/10 in Phase I to RPE 5–6/10 in Phase II and RPE 6–7/10 in Phases III and IV, with detailed weekly exercise content, training parameters, dosage, phase-specific intensity and progression criteria, and permitted exercise modifications provided in Supplementary File S1.

#### 2.8.3. Session Structure

Each 60-min POSH-Ex session will follow a standardised three-part structure comprising a 5-min warm-up, a 50-min main therapeutic exercise component, and a 5-min cool-down. The content and complexity of the exercises within each component will vary according to the corresponding intervention phase and the participant’s individual progression.

The 5-min warm-up will prepare participants for the main therapeutic component through breathing, mobility, body awareness, and low-intensity movement exercises. The 50-min main therapeutic component will incorporate phase-specific exercises targeting pelvic floor muscle function, lumbopelvic control, trunk stability, functional strength, postural control, and movement integration. The 5-min cool-down will focus on gradual reduction in exercise intensity, breathing regulation, mobility, and relaxation.

Throughout each session, emphasis will be placed on movement quality, coordinated breathing, appropriate pelvic floor muscle activation and relaxation, regulation of intra-abdominal pressure, and symptom-free exercise performance. Exercise intensity and complexity will be individually adjusted according to the participant’s functional capacity, movement quality, and symptom response.

#### 2.8.4. Safety and Exercise Modification

Participant safety will be monitored throughout the 12-week POSH-Ex intervention. During supervised sessions, the physiotherapist will continuously observe exercise performance, movement quality, and participants’ responses to exercise. Participants will also be instructed to report any pain, urinary leakage, pelvic heaviness or pressure, musculoskeletal discomfort, excessive fatigue, dizziness, or any other new or worsening symptoms occurring during or following exercise.

Exercise modifications will be individualised according to each participant’s functional capacity, movement quality, and symptom response. When required, modifications may include reducing exercise intensity or complexity, decreasing the range of motion, reducing external resistance, modifying the number of repetitions or duration of an exercise, providing additional rest periods, or temporarily replacing or discontinuing an exercise. Progression will only occur when exercises can be performed with appropriate breathing, pelvic floor muscle coordination, and lumbopelvic control and without clinically relevant adverse symptoms.

Any adverse symptoms or events occurring during the intervention will be documented by the supervising physiotherapist, together with their timing, severity, relationship to exercise, and any modifications or actions undertaken. If symptoms suggest that continued participation in exercise may not be safe, the affected exercise or session will be discontinued, and the participant will be advised to seek appropriate medical assessment. Resumption or further modification of the intervention will be determined according to symptom resolution and the participant’s clinical status.

#### 2.8.5. Intervention Fidelity and Adherence

Intervention fidelity will be maintained through standardisation of the POSH-Ex programme with respect to exercise content, session structure, weekly progression, prescribed dosage, verbal instructions, illustrated exercise materials, progression criteria, and permitted exercise modifications.

Adherence will be monitored throughout the 12-week intervention using physiotherapist attendance records and participant exercise diaries. Each participant will be prescribed a total of 24 exercise sessions. Physiotherapist-supervised sessions and independently completed replacement sessions will be recorded separately. Overall adherence will be calculated as the number of completed sessions divided by the 24 prescribed sessions and expressed as a percentage. The proportion of sessions completed under direct physiotherapist supervision will additionally be reported separately.

To ensure regular professional supervision, participants will be required to complete at least one of the two weekly sessions under synchronous physiotherapist supervision. When a scheduled supervised session is missed, the participant will be instructed to complete the corresponding session independently within the same week using the standardised exercise materials provided. Completion of independently performed replacement sessions will be self-recorded in the exercise diary. For the per-protocol analysis, participants in the intervention group will be considered adherent if they complete at least 80% of the 24 prescribed exercise sessions (≥20 sessions), including at least 10 sessions completed under direct physiotherapist supervision during the 12-week intervention.

#### 2.8.6. Control Group

Participants allocated to the control group will receive usual postpartum care and will not receive the POSH-Ex intervention or any other structured exercise programme provided as part of the study during the 12-week study period. In the context of this study, usual postpartum care refers to routine postpartum healthcare available through the Croatian healthcare system, including routine postnatal medical care and community-based postnatal support, as clinically indicated. The study will not alter, restrict, or standardise the healthcare received by participants outside the trial. Participants will continue their usual daily activities, and no restrictions will be imposed on their habitual physical activity or usual daily routines.

Participants in the control group will undergo the same assessment schedule as participants in the intervention group. All study outcomes will be assessed at baseline (T0) and reassessed after 12 weeks (T1) using the same standardised questionnaires administered to the intervention group. This will enable between-group comparisons of changes in sexual function, physical activity, fatigue, and depressive symptoms over the 12-week study period.

Any initiation of a structured exercise, physiotherapy, or rehabilitation programme outside the study in either group during the 12-week period will be documented as a potential co-intervention. Participation in such activities will not automatically result in exclusion from the study but will be considered when interpreting the study findings and the potential for contamination between groups.

### 2.9. Measures

At baseline, participant characteristics will be collected using a study-specific questionnaire. The questionnaire will collect sociodemographic, anthropometric, obstetric, and postpartum characteristics, including age, body mass and height for the calculation of body mass index (BMI), parity, mode of delivery, breastfeeding status, and episiotomy. Partner and family support will also be assessed using seven study-specific questions addressing the availability of support, its approximate duration during the day, and the type of support provided. These data will be used to characterise the study population and, where appropriate, selected variables will be considered as potential covariates in the statistical analyses.

#### 2.9.1. Primary Outcome: Sexual Function

The primary outcome will be female sexual function at the 12-week assessment (T1), assessed using the Croatian version of the Female Sexual Function Index (FSFI), with baseline FSFI total score included as a covariate in the primary analysis. The FSFI is a 19-item self-report instrument assessing sexual function during the preceding four weeks across six domains: desire, arousal, lubrication, orgasm, satisfaction, and pain. The Croatian version was developed and validated by Milić Vranješ et al. [36]. Domain scores are combined to generate a total score ranging from 2 to 36, with higher scores indicating better sexual function. A total score below 26.55 is commonly used as an indicator of possible female sexual dysfunction, although interpretation of this threshold requires consideration of sexual activity status [37,38].

The primary endpoint will be the between-group difference in FSFI total score at T1, adjusted for baseline FSFI total score. Changes in individual FSFI domain scores will be examined as secondary outcomes.

Because several FSFI items are assigned a score of zero when no sexual activity or intercourse is reported during the preceding four weeks, sexual inactivity may result in low total scores that should not automatically be interpreted as indicating sexual dysfunction [37,39]. Participants’ sexual activity status during the FSFI reference period will therefore be documented at both T0 and T1 and treated as an important contextual variable for interpretation of the FSFI total score. Baseline sexual activity status will not be used as a stratification factor during randomisation but will be summarised descriptively by study group to allow assessment of any clinically relevant baseline imbalance. Participants will be classified according to whether they report sexual activity during the relevant four-week reference period. Changes in sexual activity status between T0 and T1 will be considered separately from changes in sexual function, as an increase in the FSFI total score may partly reflect the resumption of sexual activity rather than an improvement in sexual function among participants who were already sexually active. The primary analysis will use the FSFI total score according to the standard scoring procedure; however, the established cut-off score of 26.55 will not be automatically interpreted as indicating sexual dysfunction in participants reporting no sexual activity during the relevant reference period. To assess the robustness of the primary findings, a sensitivity analysis will additionally be conducted among participants reporting sexual activity during the relevant assessment periods.

#### 2.9.2. Secondary Outcomes

Secondary outcomes will include physical activity, fatigue, and depressive symptoms assessed at baseline (T0) and at the 12-week assessment (T1). All secondary outcomes will be assessed using validated self-administered questionnaires administered at both assessment time points.

Physical activity will be assessed using the validated Croatian version of the International Physical Activity Questionnaire—Short Form (IPAQ-SF), a self-report questionnaire assessing physical activity performed during the preceding seven days [40,41,42]. The IPAQ-SF assesses walking, moderate-intensity physical activity, vigorous-intensity physical activity, and time spent sitting. Physical activity will be expressed as metabolic equivalent task minutes per week (MET-min/week) and calculated according to the standard IPAQ scoring protocol [43]. Total physical activity and activity-specific scores will be derived, with higher MET-min/week values indicating greater levels of physical activity. The secondary physical activity outcomes will be the between-group differences in total physical activity and individual physical activity domain scores at T1, adjusted for their corresponding baseline values. At T1, participants will report all physical activity performed during the preceding seven days in accordance with the standard IPAQ-SF instructions, including activity undertaken as part of the POSH-Ex programme where applicable. Accordingly, the T1 IPAQ-SF score will reflect overall physical activity during the reference period rather than physical activity performed independently of the intervention. Any increase in IPAQ-SF scores in the intervention group will therefore be interpreted cautiously, as it may partly reflect physical activity performed as part of the POSH-Ex programme and will not be considered evidence of an increase in habitual physical activity independent of the intervention. The same questionnaire administration and scoring procedures will be applied in both study groups at T0 and T1.

Fatigue will be assessed using the Fatigue Assessment Scale (FAS), a 10-item self-report instrument developed to assess the overall experience of fatigue [44]. Each item is rated on a five-point Likert scale, and after reverse-scoring the positively worded items, scores are summed to produce a total score ranging from 10 to 50, with higher scores indicating greater fatigue. Scores below 22 indicate a normal level of fatigue, scores from 22 to 34 indicate mild-to-moderate fatigue, and scores of 35 or higher indicate severe fatigue [45]. The FAS is interpreted primarily as a unidimensional measure of fatigue and has demonstrated satisfactory psychometric properties in postpartum women, including good internal consistency (Cronbach’s α = 0.80) [46]. The secondary fatigue outcome will be the between-group difference in FAS total score at T1, adjusted for baseline FAS total score.

Postpartum depressive symptoms will be assessed using the validated Croatian version of the Edinburgh Postnatal Depression Scale (EPDS), a 10-item self-report screening instrument developed to identify women at increased risk of depression during the postpartum period [47,48]. The EPDS assesses emotional experiences during the preceding seven days, with each item scored from 0 to 3, yielding a total score ranging from 0 to 30; higher scores indicate greater severity of depressive symptoms. A total score of ≥10 will be considered indicative of elevated depressive symptoms requiring further assessment, while a score of ≥13 will indicate a higher probability of clinically significant depression [47,48]. The EPDS is a screening instrument and will not be used to establish a clinical diagnosis of depression. EPDS scores will be reviewed by the principal investigator. At baseline (T0), responses will be reviewed before the participant proceeds to randomisation. At the post-intervention assessment (T1), responses will be reviewed before the follow-up assessment is considered complete. Participants with elevated EPDS scores (≥10) will be informed of the screening result and advised to seek further assessment from an appropriate healthcare professional. Particular attention will be given to item 10, which assesses thoughts of self-harm. Any response other than “never” to item 10 will prompt direct contact with the participant by the principal investigator. At T0, this contact will occur before randomisation. If direct contact cannot be established, at least two contact attempts will be made using the contact details provided for study communication. At T0, a participant with a positive response to item 10 will not proceed to randomisation until direct contact has been established. During contact, the participant will be informed of the screening result and advised to seek prompt professional assessment. If the participant indicates an immediate risk of self-harm, she will be advised to seek immediate emergency medical assistance. Elevated EPDS scores alone will not constitute an exclusion criterion or result in automatic discontinuation from the study. The Croatian version of the EPDS has demonstrated satisfactory psychometric properties, including high internal consistency (Cronbach’s α = 0.86), while the EPDS has demonstrated good diagnostic accuracy for detecting major depression in pregnant and postpartum women [48,49]. The secondary depressive-symptom outcome will be the between-group difference in EPDS total score at T1, adjusted for baseline EPDS total score. The prevalence of scores above the predefined screening thresholds will additionally be reported descriptively. EPDS scores will also be included in exploratory analyses examining their relationships with physical activity, fatigue, and sexual function.

### 2.10. Assessment Schedule

Intervention adherence, attendance at supervised sessions, independently completed replacement sessions, exercise modifications, and intervention-related adverse events will be monitored throughout the 12-week intervention period. Potential co-interventions initiated during the study period will also be documented in both study groups. The schedule of enrolment, intervention, and outcome assessments is presented in the SPIRIT schedule (Table 2), developed in accordance with the SPIRIT recommendations [50,51].

### 2.11. Data Collection and Management

Study outcome data will be collected primarily using electronic self-administered questionnaires administered through Google Forms. As configured for this study, Google Forms automatically collects participants’ email addresses when questionnaires are submitted electronically. Email addresses are collected solely for participant identification and study-related communication, including the distribution of study information and questionnaires, follow-up communication, and direct contact when required for participant safety, including the safety procedure related to EPDS item 10. Participants who are unable or prefer not to complete the questionnaires electronically will be offered equivalent paper-based versions containing identical content and standardised instructions. The principal investigator will review completed questionnaires for completeness and consistency before their inclusion in the study database.

Electronic questionnaires will be administered using Google Forms through the official institutional Google Workspace account of University North. Email addresses will be automatically collected through the study configuration and used solely for participant identification and study-related communication, including direct contact when required for participant safety. Electronic responses submitted through Google Forms will be automatically timestamped and subsequently exported to a password-protected and encrypted computer accessible only to the principal investigator. Data obtained using paper-based questionnaires will be entered into the study database using the same predefined coding system and verified against the original questionnaires.

Each participant will be assigned a unique participant identification code at enrolment. Questionnaire data will be pseudonymised before inclusion in the analytical dataset, and only pseudonymised data will be used for statistical analysis. Email addresses and other directly identifying information will be stored separately from the pseudonymised research dataset and will not be included in the analytical dataset. The document linking participant identities and contact information to their unique identification codes will also be stored separately from the research database and will be accessible only to the principal investigator.

Data quality will be ensured through verification of completeness and consistency before analysis. Any discrepancies or incomplete records will be reviewed against the original source data whenever possible.

All study data will be handled confidentially and processed in accordance with applicable data protection requirements, including the General Data Protection Regulation (GDPR). The study database and the separately stored participant identification and linkage information will be retained for five years following completion of the study, after which identifiable information will be securely destroyed.

### 2.12. Sample Size Calculation

The sample size was estimated a priori based on the primary outcome of sexual function, assessed using the FSFI. Because no previous randomised trial has evaluated an intervention equivalent to POSH-Ex, the target difference could not be derived directly from a comparable trial and was therefore specified on the basis of the best available comparative evidence and clinical judgement [52]. A meta-analysis of five trials of pelvic floor muscle training (PFMT) in postpartum women reported a pooled standardised mean difference in sexual function of 0.46 (95% CI 0.12–0.81) in favour of PFMT, with substantial heterogeneity between trials (I^2^ = 68%) [14], whereas a more recent meta-analysis identified only two relevant randomised trials and rated the evidence for sexual function as of very low certainty [30]. Because POSH-Ex combines pelvic floor muscle activation and relaxation with breathing, trunk stabilisation, functional strengthening, and movement re-education, and is delivered as 24 physiotherapist-led sessions, an effect larger than that of PFMT alone was considered plausible, although not certain. A moderate-to-large standardized effect size (Cohen’s *d* = 0.70) was therefore specified as the target difference. This value lies within the 95% confidence interval of the pooled PFMT estimate and was considered by the investigators to represent the smallest effect that would justify the time and resources required from participants and healthcare services for a 12-week supervised programme. Because this assumption may be optimistic, its implications for statistical power are described below.

The sample size calculation was performed using G*Power version 3.1 [53] for a two-tailed independent-samples *t*-test, with a significance level of α = 0.05, statistical power of 80%, and a 1:1 allocation ratio. Under these assumptions, a minimum total sample of 68 participants (34 per group) with evaluable primary outcome data was required (actual power, 81.2%). To allow for an anticipated attrition rate of approximately 20%, the target sample size was set at 84 randomised participants (42 per group); with 20% attrition (approximately 67 evaluable participants), the power of the unadjusted comparison would remain approximately 80% (80.6%). The target of 84 participants therefore represents a pragmatic allowance for attrition with equal allocation rather than the exact result of inflating the required sample by 20%.

**Figure 1:**
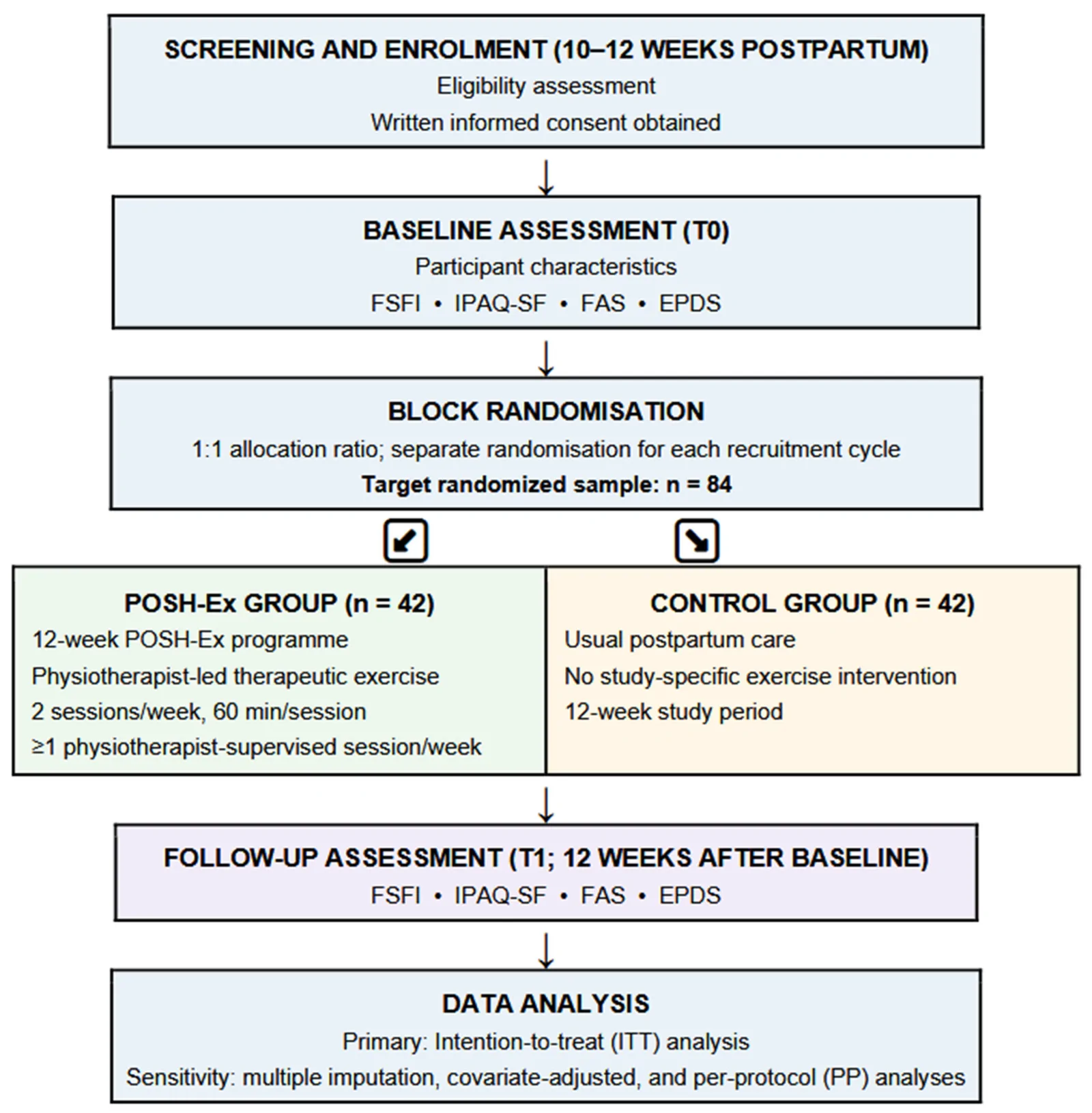
Planned CONSORT flow diagram of the POSH-Ex randomized controlled trial.

The sample size was calculated for an unadjusted between-group comparison, whereas the primary analysis will use ANCOVA adjusted for baseline FSFI total score. Adjustment for a baseline measurement that is correlated with the outcome reduces the residual variance by a factor of approximately (1 − *ρ*^2^), where *ρ* is the correlation between baseline and follow-up scores; consequently, an ANCOVA requires approximately (1 − *ρ*^2^) times the number of participants required for a *t*-test to achieve the same power [54]. The *t*-test-based calculation is therefore equivalent to assuming *ρ* = 0 and represents a conservative approach. Because empirical estimates of the correlation between FSFI scores measured 12 weeks apart in postpartum women are limited, and because this correlation may be attenuated by the resumption of sexual activity during the study period, a moderate correlation of *ρ* = 0.50 was assumed to illustrate the expected gain in precision. Under this assumption, 68 evaluable participants would provide approximately 90% power to detect *d* = 0.70 with the planned ANCOVA, or 80% power to detect a smaller effect of *d* = 0.60. The trial would, however, have limited power (approximately 58%) to detect an effect of the magnitude reported for PFMT alone (*d* = 0.46). The planned flow of participants through recruitment, randomisation, allocation, follow-up, and analysis is presented in Figure 1.

### 2.13. Statistical Analysis

Statistical analyses will be performed using IBM SPSS Statistics version 29.0 (IBM Corp., Armonk, NY, USA). All statistical tests will be two-tailed, with statistical significance set at *p* < 0.05.

Descriptive statistics will be used to summarise participant characteristics and outcome measures. Continuous variables will be presented as means and standard deviations or medians and interquartile ranges, as appropriate, while categorical variables will be presented as frequencies and percentages. Distributional characteristics of continuous variables will be assessed using the Shapiro–Wilk test together with visual inspection of histograms and Q–Q plots. For ANCOVA models, assumptions will additionally be assessed by examining the linearity of the relationship between the baseline covariate and the corresponding T1 outcome, homogeneity of regression slopes across treatment groups, homoscedasticity and distribution of residuals, and the presence of potentially influential observations or outliers. Baseline characteristics will be summarised by study group; formal statistical testing of baseline characteristics will not be used to assess the success of randomisation.

The FSFI total score will constitute the single primary outcome. Total physical activity assessed using the IPAQ-SF, fatigue assessed using the FAS total score, and depressive symptoms assessed using the EPDS total score will constitute prespecified principal secondary outcomes. FSFI domain scores and individual IPAQ-SF activity domains will be treated as additional supportive secondary outcomes, while analyses of associations between changes in outcome measures will be considered exploratory. Because the trial has a single primary outcome and a single primary comparison, no adjustment for multiplicity will be applied to the primary analysis. The primary analysis will evaluate the effect of the POSH-Ex intervention on sexual function at T1 using ANCOVA. The FSFI total score at T1 will be the dependent variable, treatment group (POSH-Ex vs. control) will be included as the fixed factor, and baseline FSFI total score will be included as a covariate. The intervention effect will be reported as the adjusted between-group mean difference in FSFI total score at T1, together with the corresponding 95% confidence interval and *p*-value. Unadjusted descriptive changes from T0 to T1 within each group will also be reported to facilitate clinical interpretation.

The same baseline-adjusted ANCOVA approach will be applied to continuous secondary outcomes, with the corresponding T1 outcome as the dependent variable and its baseline value included as a covariate. Individual IPAQ-SF activity domains and FSFI domain scores will be examined in additional secondary analyses using the same baseline-adjusted approach. Adjusted between-group mean differences and corresponding 95% confidence intervals will be reported. Secondary and domain-level analyses will be considered supportive and will be interpreted cautiously in the context of multiple comparisons; no confirmatory conclusions will be based solely on nominal statistical significance in these analyses. Changes in IPAQ-SF outcomes will be interpreted in the context of the structured exercise included in the POSH-Ex intervention; therefore, an increase in IPAQ-SF scores in the intervention group will not be interpreted as necessarily indicating an increase in habitual physical activity independent of the intervention. Non-program physical activity will not be analysed separately because the IPAQ-SF does not allow study-related exercise to be reliably distinguished from other physical activity performed during the same recall period.

The primary analysis population will be defined according to the intention-to-treat (ITT) principle, with participants analysed according to their originally allocated group. The amount and pattern of missing outcome data will be examined and reported by the study group. The primary ANCOVA will be based on participants with the observed data required for the model; therefore, this analysis represents an available-case analysis within the ITT-defined population. This analysis provides valid estimates if missingness of the 12-week FSFI total score depends only on the variables included in the model (treatment group and baseline FSFI total score), but it does not by itself include all randomised participants. All randomised participants will therefore be retained in the ITT population irrespective of adherence to the allocated intervention, and follow-up questionnaires will be requested from all participants, including those who discontinue the intervention, unless they withdraw consent.

To assess the robustness of the primary findings to missing outcome data and to more fully reflect the ITT principle, a prespecified sensitivity analysis will be conducted using multiple imputation by chained equations (fully conditional specification) under a missing-at-random (MAR) assumption [55]. Under this assumption, the probability that an outcome is missing may depend on observed data but not on the unobserved outcome value itself. Imputation will be performed separately within each randomised group so that the imputation model allows for differences between groups in the relationships among variables. The imputation model will include the baseline and 12-week values of the primary and principal secondary outcomes (FSFI total score, IPAQ-SF total physical activity, and FAS and EPDS total scores), sexual activity status at T0 and T1, the prespecified baseline covariates used in the covariate-adjusted sensitivity analysis (recruitment cycle, breastfeeding status, mode of delivery and episiotomy, and parity), and age and BMI as auxiliary variables potentially associated with missingness or with the outcome. Missing continuous variables will be imputed using predictive mean matching and missing binary variables using logistic regression. Fifty imputed datasets will be generated, which exceeds the commonly recommended minimum of one imputation per percentage of participants with incomplete data [55], and convergence of the imputation algorithm will be checked graphically. The primary ANCOVA model, fitted as a linear regression model including the treatment group and baseline FSFI total score, will be estimated in each imputed dataset, and the estimates will be combined using Rubin’s rules [56] to obtain a pooled adjusted between-group mean difference with its 95% confidence interval and *p*-value. Because the MAR assumption cannot be verified from the observed data, reasons for missing outcome data will be recorded whenever possible, and the results of the primary and multiple-imputation analyses will be interpreted together in light of the amount and pattern of missing data.

A per-protocol (PP) analysis will be conducted as a secondary sensitivity analysis. The PP population will include participants with the outcome data required for the analysis who complete the study without major protocol deviations. The intervention is designed to include at least one supervised session per week; however, for the purpose of defining the PP population, adherence will be based on the prespecified cumulative threshold of at least 20 of the 24 prescribed sessions, including at least 10 physiotherapist-supervised sessions. Weekly supervised participation in every intervention week will therefore not constitute an additional PP eligibility criterion. In the control group, participation in additional structured exercise, rehabilitation, or physiotherapy during the study period will be documented, and only deviations prespecified as major and considered sufficient to compromise adherence to the assigned control condition will result in exclusion from the PP population. Major protocol deviations relevant to the PP analysis will be defined before database lock and before group identities are revealed to the statistician.

Because sexual inactivity during the FSFI reference period may result in low total scores that do not necessarily indicate sexual dysfunction, FSFI total scores will be calculated according to the standard scoring procedure irrespective of sexual activity status in the primary analysis. Sexual activity status at T0 and T1 will be considered an important contextual variable when interpreting the primary outcome, and changes in sexual activity status will be considered separately from changes in sexual function. A prespecified sensitivity analysis will additionally evaluate the primary outcome among participants reporting sexual activity during the relevant FSFI reference periods. The established FSFI cut-off score of 26.55 will not be automatically interpreted as indicating sexual dysfunction among participants reporting no sexual activity during the relevant reference period.

As an additional prespecified sensitivity analysis, the primary ANCOVA model will be further adjusted for a limited set of baseline covariates selected a priori to assess the robustness of the estimated intervention effect to chance imbalances in prognostic factors. The covariates were selected on the basis of their reported associations with postpartum sexual function and their clinical relevance, and because they are measured before randomisation and cannot be affected by the intervention [9,26,57]: breastfeeding status at baseline (yes/no); mode of delivery and perineal trauma, combined into a single variable (caesarean birth; vaginal birth without episiotomy; vaginal birth with episiotomy); parity (primiparous/multiparous); and sexual activity status during the baseline FSFI reference period (yes/no). Recruitment cycle, the stratification factor used in randomisation, will also be included, because omitting a stratification factor from the analysis does not bias the estimated treatment effect but results in overly conservative inference [58]. The number of covariates was deliberately restricted to limit the number of model parameters relative to the expected number of participants with evaluable outcome data and thereby to avoid unstable estimates [59]; if any covariate category includes fewer than approximately 10% of participants, categories will be combined (e.g., vaginal vs. caesarean birth) before the analysis and without reference to outcome data. Age and BMI will be summarised descriptively and used as auxiliary variables in the multiple imputation model but will not be included as adjustment covariates, in order to preserve model parsimony. Partner and family support, assessed using study-specific questions without an established summary score, will not be included as a covariate. Associations of breastfeeding status, mode of delivery and episiotomy, parity, and partner and family support with FSFI scores will be examined in exploratory analyses; because the trial is not powered to detect effect modification, any analyses of whether these characteristics modify the intervention effect will be considered hypothesis-generating.

Exploratory associations between changes in sexual function and changes in total physical activity, fatigue, and depressive symptoms from T0 to T1 will be examined using Pearson or Spearman correlation coefficients, according to the distributional characteristics of the variables, and will be reported with 95% confidence intervals without adjustment for multiplicity. These association analyses will be considered exploratory and will not be used to draw confirmatory conclusions regarding intervention effectiveness.

The statistical analysis plan will be finalised before database lock and before the independent statistician is provided with the key linking the coded treatment groups to their actual group identities. Statistical analyses will be conducted by an independent statistician blinded to group identity.

## 3. Discussion

The POSH-Ex Study is designed to determine whether a structured, progressive, physiotherapist-led therapeutic exercise programme can improve sexual function in women during the early postpartum period. Previous non-pharmacological interventions targeting postpartum sexual function have predominantly focused on individual therapeutic modalities, particularly pelvic floor muscle training, with some studies reporting favourable effects on sexual function and related symptoms [14,60,61]. However, the available evidence remains heterogeneous with respect to intervention content, duration, supervision, and outcome assessment, limiting direct comparison between studies and the development of standardised rehabilitation approaches [14,60]. In contrast, POSH-Ex combines pelvic floor muscle training with breathing and relaxation exercises, trunk stabilisation, progressive functional strengthening, flexibility, movement re-education, and functional integration within a standardised 12-week programme. By integrating these components within a single physiotherapist-led intervention and positioning sexual function as the primary outcome, the present trial is designed to determine whether a standardised, multimodal therapeutic exercise programme can improve postpartum sexual function compared with usual postpartum care.

The theoretical rationale for POSH-Ex is based on a multidimensional understanding of postpartum recovery. Sexual function following childbirth is influenced by interacting neuromuscular, biomechanical, psychological, hormonal, and social factors rather than by pelvic floor muscle function alone [57]. Pregnancy and childbirth may be accompanied by alterations in pelvic floor muscle function, lumbopelvic control, motor control, and movement patterns, which may affect functional recovery and sexual well-being [60]. Accordingly, POSH-Ex extends beyond isolated pelvic floor muscle training by integrating pelvic floor muscle activation and relaxation with breathing coordination, trunk stabilisation, progressive functional strengthening, flexibility, and movement re-education. The multimodal design is intended to test whether simultaneously addressing several potentially relevant physical and functional components of postpartum recovery results in a clinically meaningful improvement in sexual function. Importantly, the present trial will allow this proposed benefit to be evaluated empirically rather than assuming that improvements in pelvic floor muscle function alone translate into improved overall sexual function.

Beyond sexual function, the present trial will examine physical activity, fatigue, and depressive symptoms as interrelated dimensions of postpartum recovery. This broader assessment is relevant because sexual health after childbirth does not develop independently of women’s physical and psychological well-being. Previous evidence has shown that postpartum exercise may contribute to reductions in depressive symptoms and improvements in psychological well-being [11,12], while higher levels of physical activity have been associated with lower perceived fatigue among postpartum women [19]. Furthermore, postpartum fatigue and depressive symptoms are closely interrelated and may influence women’s daily functioning, motivation, and engagement in intimate relationships [5]. By evaluating these outcomes alongside sexual function within the same trial, the POSH-Ex Study may provide a more comprehensive understanding of whether changes in sexual health occur in parallel with broader changes in physical and psychological recovery. Interpretation of changes in physical activity will require particular caution because exercise performed as part of the POSH-Ex programme may contribute to participants’ IPAQ-SF scores at follow-up. Consequently, higher IPAQ-SF scores in the intervention group may partly reflect physical activity prescribed within the intervention and will not necessarily indicate an increase in habitual physical activity independent of POSH-Ex. Exploratory analyses of relationships among these outcomes may additionally identify clinically relevant patterns for future investigation; however, such findings will be interpreted cautiously and will not be considered evidence of causal or mediating mechanisms.

A further distinguishing feature of POSH-Ex is the combination of a standardised intervention structure with synchronous online physiotherapist supervision. Detailed specification of intervention components, dosage, progression criteria, and permitted modifications is consistent with recommendations for transparent reporting and reproducibility of exercise-based interventions [34,35]. The online delivery format may additionally reduce geographical, logistical, and time-related barriers to participation, which may be particularly relevant during the postpartum period, while synchronous supervision allows real-time physiotherapist feedback, exercise modification, and monitoring of exercise performance and safety. Previous evidence indicates that postpartum telehealth exercise interventions vary considerably in their design and delivery, with relatively few incorporating real-time videoconferencing or supervision by qualified exercise professionals [62]. By combining structured progression with synchronous professional supervision, the present trial will evaluate POSH-Ex under defined and standardised study conditions. However, feasibility, acceptability, cost-effectiveness, implementation, and scalability are not evaluated as outcomes in the present trial; therefore, the findings will not establish whether POSH-Ex can be implemented effectively in routine clinical practice. These aspects will require evaluation in future implementation-focused research.

If effective, POSH-Ex could have relevant implications for the role of physiotherapy within multidisciplinary postpartum care. Sexual health is an integral component of overall health and well-being, yet it remains insufficiently addressed within routine postpartum care and rehabilitation [57,63]. If a clinically meaningful benefit is demonstrated, POSH-Ex may provide a structured framework for further investigation of physiotherapist-led approaches to postpartum sexual health while simultaneously considering physical activity, fatigue, and psychological well-being. Such findings could also inform future research examining how structured physiotherapy interventions might be incorporated within multidisciplinary postpartum care pathways. However, the present trial is designed primarily to evaluate the effectiveness of POSH-Ex; therefore, its integration into routine postpartum care should be considered only after its effects have been established and implementation has been evaluated in broader clinical settings.

The POSH-Ex study has several methodological strengths, including randomized group allocation, an a priori sample-size calculation, a standardised and progressively structured 12-week intervention, predefined adherence and progression criteria, prospective monitoring of safety and co-interventions, the use of established validated outcome measures, and statistical analysis conducted by an independent statistician blinded to group identity. Nevertheless, several limitations should be acknowledged. Owing to the nature of the intervention, participants and the treating physiotherapist cannot be blinded, which may introduce performance- and expectation-related bias. In addition, the primary and secondary outcomes are based on self-reported measures and may therefore be susceptible to recall and reporting bias. In particular, physical activity assessed using the IPAQ-SF may be subject to overestimation, including overreporting of moderate-to-vigorous physical activity, and the instrument does not permit reliable separation of POSH-Ex-related activity from other physical activity reported within the same recall period. Sexual function during the postpartum period may also be influenced by factors that cannot be fully controlled within the trial, including breastfeeding, perineal trauma, partner support, resumption of sexual activity, and initiation of other exercise or physiotherapy interventions. In particular, sexual activity status requires careful consideration when interpreting FSFI scores, as participants who report no sexual activity during the four-week reference period may obtain lower scores because of the standard FSFI scoring procedure. Moreover, resumption of sexual activity during the 12-week study period may contribute to changes in FSFI scores independently of changes in sexual function among women who were already sexually active. This issue will be addressed through consideration of sexual activity status at both assessment points and a prespecified sensitivity analysis among sexually active participants. Breastfeeding status, mode of delivery, perineal trauma, parity, and partner support may also contribute to heterogeneity in postpartum recovery and sexual function and should therefore be considered when interpreting variation in study outcomes. Given the planned sample size, however, including all potentially relevant maternal and postpartum characteristics simultaneously as covariates would risk unstable model estimates; the primary analysis will therefore be adjusted for baseline FSFI only, while breastfeeding status, mode of delivery and episiotomy, parity, and baseline sexual activity status will be included in a prespecified covariate-adjusted sensitivity analysis. Although these factors will be documented where relevant, residual confounding cannot be excluded. Furthermore, online delivery requires adequate internet access, digital literacy, and willingness to participate in remotely supervised exercise, which may limit the external validity and generalisability of the findings to postpartum women with different socioeconomic or technological circumstances or those who prefer face-to-face care. Although the standardised online format and synchronous supervision may support intervention fidelity under the conditions of the trial, these same requirements may limit the applicability of the findings to broader postpartum populations and healthcare settings. The 12-week follow-up will allow assessment of short-term post-intervention outcomes but will not determine whether any observed changes in sexual function, physical activity, fatigue, or depressive symptoms are sustained over the longer term. This is particularly relevant because postpartum recovery is dynamic, and sexual function may continue to change beyond the intervention period. Longer-term follow-up will therefore be needed to evaluate the durability of any observed effects. An additional limitation is the absence of a sham or attention-matched control condition. Because the control group will receive usual postpartum care without a study-specific exercise intervention, any between-group difference may reflect not only the physiological effects of the therapeutic exercise programme but also non-specific effects associated with physiotherapist attention, structured contact, social interaction, expectations, or increased motivation. The trial therefore evaluates the effect of adding POSH-Ex to usual postpartum care rather than isolating the specific physiological effects of exercise alone. Adherence and intervention exposure will also be important when interpreting the trial results. The extent of adherence and intervention exposure will therefore be considered when interpreting the primary findings, particularly if no statistically significant between-group difference is observed. However, the per-protocol analysis will remain a secondary sensitivity analysis, as analyses based on post-randomisation adherence may be susceptible to selection bias and will not replace the primary analysis. In addition, the sample size was based on an assumed moderate-to-large effect (*d* = 0.70); smaller but potentially clinically relevant effects, such as those reported for pelvic floor muscle training alone, may therefore not be detected with adequate power, and a non-significant result should be interpreted in light of the confidence interval for the between-group difference rather than as evidence of no effect. Despite these limitations, the trial is designed to evaluate a standardised, multimodal, physiotherapist-led approach to postpartum sexual health and may inform the design of future larger-scale studies with longer follow-up.

## 4. Conclusions

This protocol describes the rationale and methodology of the POSH-Ex randomised controlled trial, designed to evaluate whether a standardised, multimodal, physiotherapist-led therapeutic exercise programme improves sexual function in women during the early postpartum period compared with usual postpartum care. The trial will also assess selected physical and psychological dimensions of postpartum recovery as secondary outcomes. If POSH-Ex demonstrates a clinically meaningful benefit, the findings may inform future research on structured physiotherapy approaches that recognise sexual health as an integral component of comprehensive postpartum care.

## Figures and Tables

**Table 1:** Conceptual Framework and Progression of the POSH-Ex Intervention.

Phase	Weeks	Training Focus	Main Exercise Components	Progression Principle
Phase I: Neuromuscular regulation and PFM awareness	1–3	Develop awareness of PFM function, breathing coordination, and basic lumbopelvic control	Diaphragmatic breathing; PFM relaxation and activation; neuromuscular control exercises; basic trunk stabilisation; basic functional movement patterns	Development of selective and reversible PFM activation, breathing coordination, and correct movement execution
Phase II: Functional endurance and integrated lumbopelvic stability	4–6	Develop PFM endurance and integrated lumbopelvic stability	Endurance-oriented PFM training; trunk stabilisation; bilateral functional strengthening; postural control	Increased exercise duration, time under tension, and movement-control demands while maintaining coordinated breathing and appropriate regulation of intra-abdominal pressure
Phase III: Functional strengthening and dynamic lumbopelvic stability	7–9	Improve functional strength and dynamic lumbopelvic stability	Functional strengthening; unilateral lower-extremity exercises; dynamic trunk stabilisation; progressive resistance exercises	Increased motor complexity, functional loading, and coordination demands while preserving movement quality and symptom-free performance
Phase IV: Advanced functional integration and movement control	10–12	Integrate PFM function into complex and multidirectional movement patterns	Multiplanar functional exercises; coordinated movement sequences; balance tasks; weight transfer; dynamic postural control	Increased movement integration, motor complexity, balance demands, and coordination during continuous functional tasks

Abbreviation: PFM, pelvic floor muscles. Note: All sessions will comprise a standardised warm-up, main exercise component, and cool-down. The intervention will be delivered twice weekly for 12 consecutive weeks, with each session lasting 60 min. All supervised sessions will be delivered synchronously online by the same physiotherapist.

**Table 2:** SPIRIT Schedule of Enrolment, Intervention, and Assessments.

Activity/Measure	Enrolment	Baseline (T0)	12-Week Study Period	Post-Intervention (T1)
Eligibility screening	X			
Written informed consent	X			
Participant characteristics		X		
Randomisation		X		
POSH-Ex intervention			X	
Usual postpartum care/control condition			X	
FSFI		X		X
IPAQ-SF		X		X
FAS		X		X
EPDS		X		X
Intervention adherence			X	
Exercise modifications			X	
Adverse-event monitoring			X	
Co-intervention monitoring			X	

Note. X indicates the time point or study period during which the specified activity, intervention, or assessment is performed. FSFI, Female Sexual Function Index; IPAQ-SF, International Physical Activity Questionnaire–Short Form; FAS, Fatigue Assessment Scale; EPDS, Edinburgh Postnatal Depression Scale. The schedule was developed in accordance with the SPIRIT recommendations [50,51].

## Data Availability

No new data were created or analyzed in this study. Data sharing is not applicable to this article.

## Supplementary Materials

The following supporting information can be downloaded at https://www.mdpi.com/article/10.3390/healthcare14193277/s1, Supplementary File S1: Detailed 12-week POSH-Ex intervention protocol, including weekly exercise content, session structure, exercise execution, dosage, intensity and resistance, progression criteria, and permitted modifications.

## Abbreviations

The following abbreviations are used in this manuscript:

ANCOVA	Analysis of Covariance
BMI	Body Mass Index
CONSORT	Consolidated Standards of Reporting Trials
EPDS	Edinburgh Postnatal Depression Scale
FAS	Fatigue Assessment Scale
FSFI	Female Sexual Function Index
GDPR	General Data Protection Regulation
IPAQ-SF	International Physical Activity Questionnaire–Short Form
ITT	Intention-to-Treat
MET	Metabolic Equivalent of Task
MET-min/week	Metabolic Equivalent of Task Minutes per Week
PFM	Pelvic Floor Muscles
POSH-Ex	Postpartum Sexual Health Exercise
PP	Per-Protocol
RCT	Randomized Controlled Trial
SPIRIT	Standard Protocol Items: Recommendations for Interventional Trials
