# Supplementary material for: The POSH-Ex Study: Study Protocol for a Randomized Controlled Trial of a 12-Week Online Physiotherapy Intervention for Improving Postpartum Sexual Health
Source: Healthcare (Basel). 2026 Oct 2;14(19):3277. doi: 10.3390/healthcare14193277 (PMC13665170; doi:10.3390/healthcare14193277)
Supplement: Supplementary file 1 [file healthcare-14-03277-s001.zip › healthcare-4556351-supplementary.pdf]

SUPPLEMENTARY FILE S1

Detailed 12-week POSH-Ex intervention protocol, including weekly exercise content, session structure, exercise execution, dosage, intensity and resistance, progression criteria, and permitted modifications

Exercise Intensity and Phase-Specific Progression

The overall POSH-Ex programme is designed to progress within a moderate-to-high subjective exercise intensity range. During Phase I (weeks 1–3), intensity will be maintained at RPE 4–5/10 to prioritise movement quality, motor learning, proprioceptive awareness, and the establishment of appropriate neuromuscular patterns. In Phase II (weeks 4–6), intensity will progress to RPE 5–6/10, primarily through increased time under tension, training volume, functional complexity, and stabilisation demands. Pelvic floor muscle (PFM) contractions will remain submaximal, at approximately 60–70% of the participant's perceived maximum voluntary contraction. In Phases III and IV (weeks 7–12), overall exercise intensity will progress to RPE 6–7/10 as biomechanical, unilateral, balance, coordination, and functional demands increase. During Phase III, PFM contractions will progress to approximately 70–80% of perceived maximum voluntary contraction, where appropriate. Exercise intensity and progression will be individually adjusted within these predefined ranges according to movement quality, functional capacity, and symptom response.

General Progression, Modification, and Safety Principles

The POSH-Ex programme follows a standardised weekly exercise structure and prescribed dosage, while exercise progression and modification are individualised within predefined criteria. Progression to a more demanding exercise variant is permitted when the participant demonstrates appropriate movement quality, coordinated breathing and pelvic floor muscle function, adequate lumbopelvic control, and completion of the prescribed exercise without clinically relevant adverse symptoms. If these criteria are not met, the participant will continue with the preceding exercise variant or receive an appropriate modification. Modifications may include reducing exercise intensity or complexity, range of motion, external resistance, number of repetitions or exercise duration, increasing rest periods, or temporarily replacing or discontinuing an exercise. Participants will be instructed to report pain, urinary leakage, pelvic heaviness or pressure, musculoskeletal discomfort, excessive fatigue, dizziness, or other new or worsening symptoms. Exercise progression will not occur in the presence of clinically relevant adverse symptoms.

Week 1 – POSH-Ex Exercise Programme

Training Component	Functional Component	Exercise	Dosage	Primary Objective
**Warm-up (5 min)**	Dynamic warm-up	Marching in place with coordinated arm movements	2 min	Gradual whole-body activation and preparation for therapeutic exercise
	Breathing regulation	Supine diaphragmatic breathing	2 min	Establishment of a controlled diaphragmatic breathing pattern and preparation for coordination of breathing with movement
	Pelvic floor awareness	PFM relaxation coordinated with exhalation	1 min (~10 breathing cycles)	Development of conscious PFM relaxation and proprioceptive awareness in preparation for selective activation
**Main therapeutic exercise component (50 min)**	**1. Neuromuscular activation**	Selective PFM contraction and relaxation	3 × 8 repetitions; 5 s contraction / 5 s relaxation; 30 s rest between sets	Development of selective PFM activation and complete relaxation
		Supine posterior pelvic tilt	2 × 10 repetitions; 1 s hold; 30 s rest between sets	Development of pelvic control and coordination of breathing with movement
	**2. Lumbopelvic stabilization**	Supine bridge	2 × 8 repetitions; 6 s hold; 30 s rest between sets	Activation of the gluteal musculature and development of lumbopelvic stability with coordinated PFM activation

Training Component	Functional Component	Exercise	Dosage	Primary Objective
		Alternating supine leg extension above the support surface	2 × 8 repetitions per leg; 30 s rest between sets	Development of trunk and pelvic control during lower-limb movement with coordinated breathing
		Isometric hip adduction with a pillow or soft ball combined with ankle dorsiflexion	2 × 10 repetitions; 5 s contraction / 3 s relaxation; 30 s rest between sets	Activation of the hip adductors and contribution to pelvic girdle stabilization with coordinated ankle movement
	**3. Postural control**	Side-lying hip abduction	2 × 10 repetitions per side; 2 s hold; 30 s rest between sets	Activation of the hip abductors and development of lateral pelvic stability
		Seated anterior–posterior pelvic tilt	2 × 10 repetitions; 30 s rest between sets	Development of pelvic control in sitting with coordinated PFM activation and relaxation
		Seated pelvic circles with coordinated PFM contraction and relaxation	45 s in one direction + 45 s in the opposite direction	Development of pelvic mobility and proprioceptive awareness with coordinated PFM contraction and relaxation
	**4. Functional integration**	Partial squat with coordinated breathing and PFM activation	2 × 8 repetitions; 30 s rest between sets	Integration of breathing and PFM activation into a basic functional movement pattern
**Cool-down (5 min)**	Breathing regulation and recovery	Supine diaphragmatic breathing	1 min	Gradual reduction in activity intensity and promotion of relaxation
	Flexibility	Half-kneeling hip flexor stretch	30 s per side (1 min total)	Reduction of hip flexor muscle tension and maintenance of hip flexor flexibility
	Flexibility	Seated hip adductor stretch	1 min	Reduction of hip adductor muscle tension and maintenance of hip adductor flexibility
	Trunk flexibility and mobility	Supine trunk rotation stretch	30 s per side (1 min total)	Reduction of trunk muscle tension and promotion of trunk mobility
	Relaxation and recovery	Conscious whole-body relaxation	1 min	Reduction of residual muscle tension and completion of the therapeutic exercise session

Week 2 – POSH-Ex Exercise Programme

Training Component	Functional Component	Exercise	Dosage	Primary Objective
**Warm-up (5 min)**	Dynamic warm-up	Marching in place with coordinated arm movements	2 min	Gradual whole-body activation and preparation for therapeutic exercise
	Breathing regulation	Supine diaphragmatic breathing	2 min	Establishment of a controlled diaphragmatic breathing pattern and preparation for coordination of breathing with movement
	Pelvic floor awareness	PFM relaxation coordinated with exhalation	1 min (~10 breathing cycles)	Development of conscious PFM relaxation and proprioceptive awareness in preparation for selective activation
**Main therapeutic exercise component (50 min)**	**1. Neuromuscular activation**	Selective PFM contraction and relaxation	3 × 8 repetitions; 6 s contraction / 6 s relaxation; 30 s rest between sets	Improvement of selective PFM activation and increased duration of controlled contraction while maintaining complete relaxation between contractions
		Supine posterior pelvic tilt	2 × 12 repetitions; 1 s hold; 30 s rest between sets	Development of pelvic control and coordination of breathing with movement

Training Component	Functional Component	Exercise	Dosage	Primary Objective
	**2. Lumbopelvic stabilization**	Bridge with alternating foot lift	2 × 8 repetitions per leg; 30 s rest between sets	Development of lumbopelvic stability and pelvic control during a reduced base of support
		Dead Bug from a 90° hip and knee flexion position	2 × 8 repetitions per side; 30 s rest between sets	Development of trunk and pelvic control during unilateral lower-limb movement with coordinated breathing
		Isometric hip adduction with a pillow or soft ball combined with ankle dorsiflexion	2 × 10 repetitions; 6 s contraction / 6 s relaxation; 30 s rest between sets	Activation of the hip adductors and contribution to pelvic girdle stabilization with coordinated ankle movement
	**3. Postural control**	Side-lying hip abduction	2 × 10 repetitions per side; 2 s hold; 30 s rest between sets	Activation of the hip abductors and development of lateral pelvic stability
		Seated PFM activation with bilateral shoulder flexion	2 × 10 repetitions; 30 s rest between sets	Integration of PFM activation with upper-limb movement while maintaining postural control
		Seated pelvic circles with coordinated PFM contraction and relaxation	45 s in one direction + 45 s in the opposite direction	Development of pelvic mobility and proprioceptive awareness with coordinated PFM contraction and relaxation
	**4. Functional integration**	Partial squat with a hold in the lowered position, coordinated breathing, and PFM activation	2 × 8 repetitions; 3 s hold; 30 s rest between sets	Progression of breathing and PFM activation within a functional squat movement pattern
		Static split-squat hold	2 × 6 repetitions per leg; 8 s hold; 30 s rest between sets	Development of unilateral stability, pelvic control, and postural endurance
		Single-leg stance with bilateral arm elevation	2 × 30 s per leg; 30 s rest between sets	Development of balance, postural control, and pelvic stability with a reduced base of support
**Cool-down (5 min)**	Breathing regulation and recovery	Supine diaphragmatic breathing	1 min	Gradual reduction in activity intensity and promotion of relaxation
	Flexibility	Half-kneeling hip flexor stretch	30 s per side (1 min total)	Reduction of hip flexor muscle tension and maintenance of hip flexor flexibility
	Flexibility	Seated hip adductor stretch	1 min	Reduction of hip adductor muscle tension and maintenance of hip adductor flexibility
	Trunk flexibility and mobility	Supine trunk rotation stretch	30 s per side (1 min total)	Reduction of trunk muscle tension and promotion of trunk mobility
	Relaxation and recovery	Conscious whole-body relaxation	1 min	Reduction of residual muscle tension and completion of the therapeutic exercise session

Week 3 – POSH-Ex Exercise Programme

Training Component	Functional Component	Exercise	Dosage	Primary Objective
**Warm-up (5 min)**	Dynamic warm-up	Marching in place with coordinated arm movements	2 min	Gradual whole-body activation and preparation for therapeutic exercise
	Breathing regulation	Supine diaphragmatic breathing	2 min	Establishment of a controlled diaphragmatic breathing pattern and preparation for coordination of breathing with movement
	Pelvic floor awareness	PFM relaxation coordinated with exhalation	1 min (~10 breathing cycles)	Development of conscious PFM relaxation and proprioceptive awareness in preparation for selective activation

Training Component	Functional Component	Exercise	Dosage	Primary Objective
**Main therapeutic exercise component (50 min)**	**1. Neuromuscular activation**	Selective PFM contraction and relaxation	3 × 6 repetitions; 8 s contraction / 8 s relaxation; 30 s rest between sets	Progression of sustained selective PFM contraction while maintaining complete relaxation between contractions
		Rapid selective PFM contractions and relaxations	2 × 10 repetitions; 1 s contraction / 1–2 s relaxation; 30 s rest between sets	Development of rapid selective PFM activation with complete relaxation between contractions
	**2. Lumbopelvic stabilization**	Supine bridge with sustained hold and PFM activation	2 × 6 repetitions; 8 s hold; 30 s rest between sets	Development of isometric endurance and lumbopelvic stability with coordinated PFM activation
		Modified side plank with knee support	2 × 6 repetitions per side; 6 s hold; 30 s rest between sets	Development of lateral lumbopelvic stability and pelvic control
		Quadruped unilateral arm raise	2 × 6 repetitions per side; 6 s hold; 30 s rest between sets	Development of trunk and pelvic stability during unilateral upper-limb movement
	**3. Postural control**	Quadruped hip circles with the knee flexed	2 sets per side; 5 circles in each direction; 30 s rest between sets	Development of dynamic pelvic and hip control while maintaining trunk stability
		Single-leg stance with bilateral overhead arm elevation	2 × 6 repetitions per leg; 2 s hold; 30 s rest between sets	Development of dynamic balance and postural control with a reduced base of support and concurrent upper-limb movement
	**4. Functional integration**	Sit-to-stand with a mid-transition hold	2 × 8 repetitions; 3 s hold approximately halfway through the transition; 3 s controlled lowering; 30 s rest between sets	Integration of PFM activation and postural control into the functional sit-to-stand transition
		Reverse lunge with controlled lowering	2 × 8 repetitions per leg; 3 s lowering phase; 2 s hold; 30 s rest between sets	Integration of lumbopelvic control and PFM activation into a unilateral functional movement pattern
**Cool-down (5 min)**	Breathing regulation and recovery	Supine diaphragmatic breathing	1 min	Gradual reduction in activity intensity and promotion of relaxation
	Flexibility	Half-kneeling hip flexor stretch	30 s per side (1 min total)	Reduction of hip flexor muscle tension and maintenance of hip flexor flexibility
	Flexibility	Seated hip adductor stretch	1 min	Reduction of hip adductor muscle tension and maintenance of hip adductor flexibility
	Trunk flexibility and mobility	Supine trunk rotation stretch	30 s per side (1 min total)	Reduction of trunk muscle tension and promotion of trunk mobility
	Relaxation and recovery	Conscious whole-body relaxation	1 min	Reduction of residual muscle tension and completion of the therapeutic exercise session

Week 4 – POSH-Ex Exercise Programme

Training Component	Functional Component	Exercise	Dosage	Primary Objective
**Warm-up (5 min)**	Dynamic warm-up	Marching in place with coordinated arm movements	2 min	Gradual whole-body activation and preparation for therapeutic exercise
	Breathing regulation	Supine diaphragmatic breathing	2 min	Establishment of a controlled diaphragmatic breathing pattern and preparation for coordination of breathing with movement

Training Component	Functional Component	Exercise	Dosage	Primary Objective
	Pelvic floor awareness	PFM relaxation coordinated with exhalation	1 min (~10 breathing cycles)	Development of conscious PFM relaxation and proprioceptive awareness in preparation for selective activation
**Main therapeutic exercise component (50 min)**	**1. Neuromuscular activation**	Selective PFM contraction and relaxation	3 × 8 repetitions; 8 s contraction / 8 s relaxation; 30 s rest between sets	Progression of PFM endurance through an increased number of sustained contractions while maintaining complete relaxation between contractions
		Rapid selective PFM contractions and relaxations	3 × 10 repetitions; 1 s contraction / 1 s relaxation; 30 s rest between sets	Development of repeated rapid PFM activation with complete relaxation between contractions
	**2. Lumbopelvic stabilization**	Supine bridge with mini resistance band above the knees	3 × 6 repetitions; 8 s hold against band resistance; 45 s rest between sets	Progression of lumbopelvic stability with additional external resistance and controlled pelvic alignment
		Contralateral Dead Bug with a 1-kg dumbbell	2 × 8 repetitions per side; 3 s hold; 45 s rest between sets	Development of dynamic trunk and pelvic stability during contralateral limb movement with additional external load
	**3. Postural control**	Dowel-assisted hip hinge	2 × 8 repetitions; 3 s hold; 45 s rest between sets	Development of lumbopelvic control and maintenance of neutral spinal alignment during hip-hinge movement
	**4. Functional integration**	Partial squat with mini resistance band above the knees	3 × 10 repetitions; 3 s hold in the lowered position; 30 s rest between sets	Integration of PFM activation and lower-limb control into a functional squat pattern with external resistance
		Lateral lunge	2 × 8 repetitions per side; 3 s hold; 45 s rest between sets	Development of frontal-plane pelvic and lower-limb control and integration of PFM activation into a multiplanar functional movement pattern
**Cool-down (5 min)**	Breathing regulation and recovery	Supine diaphragmatic breathing	1 min	Gradual reduction in activity intensity and promotion of relaxation
	Flexibility	Standing hip flexor stretch with posterior pelvic tilt	30 s per side (1 min total)	Reduction of hip flexor muscle tension and maintenance of hip flexor flexibility
	Flexibility	Side-lunge adductor stretch	30 s per side (1 min total)	Reduction of hip adductor muscle tension and maintenance of hip adductor flexibility
	Trunk flexibility and mobility	Seated trunk rotation stretch	30 s per side (1 min total)	Reduction of trunk muscle tension and promotion of trunk mobility
	Relaxation and recovery	Conscious whole-body relaxation	1 min	Reduction of residual muscle tension and completion of the therapeutic exercise session

Week 5 – POSH-Ex Exercise Programme

Training Component	Functional Component	Exercise	Dosage	Primary Objective
**Warm-up (5 min)**	Dynamic warm-up	Marching in place with coordinated arm movements	2 min	Gradual whole-body activation and preparation for therapeutic exercise

Training Component	Functional Component	Exercise	Dosage	Primary Objective
	Breathing regulation	Supine diaphragmatic breathing	2 min	Establishment of a controlled diaphragmatic breathing pattern and preparation for coordination of breathing with movement
	Pelvic floor awareness	PFM relaxation coordinated with exhalation	1 min (~10 breathing cycles)	Development of conscious PFM relaxation and proprioceptive awareness in preparation for selective activation
**Main therapeutic exercise component (50 min)**	**1. Neuromuscular activation**	Selective PFM contraction and relaxation	3 × 8 repetitions; 10 s contraction / 10 s relaxation; 30 s rest between sets	Progression of PFM endurance through increased duration of sustained selective contractions while maintaining complete relaxation between contractions
		Rapid selective PFM contractions and relaxations	3 × 12 repetitions; 1 s contraction / 1–2 s relaxation; 30 s rest between sets	Progression of repeated rapid PFM activation while maintaining complete relaxation between contractions
	**2. Lumbopelvic stabilization**	Supine bridge with mini resistance band and alternating heel raises	3 × 6 repetitions; 8 s hold against band resistance with alternating heel raises; 45 s rest between sets	Progression of lumbopelvic stability through external resistance and alternating reduction of the base of support
		Contralateral Dead Bug with a 1-kg dumbbell	2 × 8 repetitions per side; 3 s hold; 45 s rest between sets	Development of dynamic trunk and pelvic stability during contralateral limb movement with additional external load
	**3. Postural control**	Single-leg hip hinge	2 × 8 repetitions per side; 3 s hold; 45 s rest between sets	Development of unilateral postural control, balance, and lumbopelvic stability during a hip-hinge movement pattern
	**4. Functional integration**	Partial squat with mini resistance band above the knees	3 × 10 repetitions; 5 s hold in the lowered position; 30 s rest between sets	Progression of PFM activation and lower-limb control within a resisted functional squat pattern
		Forward lunge with sustained hold	2 × 8 repetitions per side; 6 s hold; 45 s rest between sets	Development of unilateral lower-limb and pelvic control with integration of PFM activation into a functional movement pattern
**Cool-down (5 min)**	Breathing regulation and recovery	Supine diaphragmatic breathing	1 min	Gradual reduction in activity intensity and promotion of relaxation
	Flexibility	Standing hip flexor stretch with posterior pelvic tilt	30 s per side (1 min total)	Reduction of hip flexor muscle tension and maintenance of hip flexor flexibility
	Flexibility	Side-lunge adductor stretch	30 s per side (1 min total)	Reduction of hip adductor muscle tension and maintenance of hip adductor flexibility
	Trunk flexibility and mobility	Seated trunk rotation stretch	30 s per side (1 min total)	Reduction of trunk muscle tension and promotion of trunk mobility
	Relaxation and recovery	Conscious whole-body relaxation	1 min	Reduction of residual muscle tension and completion of the therapeutic exercise session

Week 6 – POSH-Ex Exercise Programme

Training Component	Functional Component	Exercise	Dosage	Primary Objective
**Warm-up (5 min)**	Dynamic warm-up	Marching in place with coordinated arm movements	2 min	Gradual whole-body activation and preparation for therapeutic exercise
	Breathing regulation	Supine diaphragmatic breathing	2 min	Establishment of a controlled diaphragmatic breathing pattern and preparation for coordination of breathing with movement
	Pelvic floor awareness	PFM relaxation coordinated with exhalation	1 min (~10 breathing cycles)	Development of conscious PFM relaxation and proprioceptive awareness in preparation for selective activation
**Main therapeutic exercise component (50 min)**	**1. Neuromuscular activation**	PFM activation in tall kneeling with bilateral overhead arm elevation	2 × 6 repetitions; 8 s hold; 30 s rest between sets	Integration of sustained PFM activation into an upright kneeling position with concurrent upper-limb movement
	**2. Lumbopelvic stabilization**	Supine bridge with mini resistance band and prolonged hold	3 × 5 repetitions; 10 s hold against band resistance; 45 s rest between sets	Progression of lumbopelvic stability and muscular endurance under sustained external resistance
		Dead Bug with prolonged hold	2 × 6 repetitions per side; 5 s hold; 45 s rest between sets	Progression of trunk and pelvic stability during sustained contralateral limb positioning
	**3. Postural control**	Single-leg hip hinge with a 1-kg dumbbell	2 × 6 repetitions per side; 3 s hold; 45 s rest between sets	Progression of unilateral postural control, balance, and lumbopelvic stability with additional external load
	**4. Functional integration**	Partial squat with mini resistance band and prolonged hold	3 × 8 repetitions; 8 s hold in the lowered position; 30 s rest between sets	Progression of PFM activation and lower-limb control through prolonged loading within a resisted functional squat pattern
		Forward lunge with sustained hold	2 × 6 repetitions per side; 8 s hold; 45 s rest between sets	Progression of unilateral lower-limb and pelvic control with sustained loading and integrated PFM activation
		Reverse lunge to single-leg balance	2 × 5 repetitions per side; 5 s hold in the reverse-lunge position followed by 3 s single-leg balance; 45 s rest between sets	Integration of PFM activation, unilateral lower-limb control, dynamic balance, and postural stability during a transitional functional movement
**Cool-down (5 min)**	Breathing regulation and recovery	Supine diaphragmatic breathing	1 min	Gradual reduction in activity intensity and promotion of relaxation
	Flexibility	Standing hip flexor stretch with posterior pelvic tilt	30 s per side (1 min total)	Reduction of hip flexor muscle tension and maintenance of hip flexor flexibility
	Flexibility	Side-lunge adductor stretch	30 s per side (1 min total)	Reduction of hip adductor muscle tension and maintenance of hip adductor flexibility
	Trunk flexibility and mobility	Seated trunk rotation stretch	30 s per side (1 min total)	Reduction of trunk muscle tension and promotion of trunk mobility
	Relaxation and recovery	Conscious whole-body relaxation	1 min	Reduction of residual muscle tension and completion of the therapeutic exercise session

Week 7 – POSH-Ex Exercise Programme

Training Component	Functional Component	Exercise	Dosage	Primary Objective
**Warm-up (5 min)**	Dynamic warm-up	Marching in place with coordinated arm movements	2 min	Gradual whole-body activation and preparation for therapeutic exercise
	Breathing regulation	Supine diaphragmatic breathing	2 min	Establishment of a controlled diaphragmatic breathing pattern and preparation for coordination of breathing with movement
	Pelvic floor awareness	PFM relaxation coordinated with exhalation	1 min (~10 breathing cycles)	Development of conscious PFM relaxation and proprioceptive awareness in preparation for selective activation
**Main therapeutic exercise component (50 min)**	**1. Neuromuscular activation**	Rapid selective PFM contractions and relaxations in standing	3 × 12 repetitions; 1 s contraction / 1 s relaxation; 30 s rest between sets	Progression of rapid selective PFM activation and complete relaxation in an upright functional position
	**2. Lumbopelvic stabilization**	Single-leg bridge	2 × 6 repetitions per leg; 2 s lifting phase; 8 s hold; 2 s lowering phase; 45 s rest between sets	Development of unilateral lumbopelvic stability and pelvic control with sustained loading
	**3. Postural control**	Single-leg hip hinge with a 2-kg dumbbell held in the contralateral hand	2 × 6 repetitions per side; 3 s lowering phase; 3 s hold; 2 s return phase; 45 s rest between sets	Progression of unilateral postural control, balance, and lumbopelvic stability with increased external load
		Split-stance trunk rotation with a 2-kg dumbbell	2 × 8 repetitions per side; 3 s rotation; 2 s hold; 3 s return phase; 45 s rest between sets	Development of postural control and thoracic rotation while maintaining pelvic stability, with coordinated PFM activation
	**4. Functional integration**	Squat with a 2-kg dumbbell held in front of the chest	3 × 8 repetitions; 3 s lowering phase; 2 s hold; 2 s return phase; 45 s rest between sets	Integration of PFM activation and lumbopelvic control into a loaded functional squat pattern
		Forward lunge	2 × 8 repetitions per leg; 3 s lowering phase; 2 s hold; 2 s return phase; 45 s rest between sets	Integration of PFM activation and unilateral lower-limb control into a dynamic forward-lunge pattern
		Lateral lunge	2 × 8 repetitions per side; 3 s lowering phase; 2 s hold; 2 s return phase; 45 s rest between sets	Integration of PFM activation and frontal-plane pelvic and lower-limb control into a lateral functional movement pattern
**Cool-down (5 min)**	Breathing regulation and recovery	Supine diaphragmatic breathing	1 min	Gradual reduction in activity intensity and promotion of relaxation
	Flexibility	Supine hip flexor stretch at the edge of the support surface	30 s per side (1 min total)	Reduction of hip flexor muscle tension and maintenance of hip flexor flexibility
	Flexibility	Quadruped adductor rock-back stretch	30 s per side (1 min total)	Reduction of hip adductor muscle tension and maintenance of hip adductor flexibility
	Trunk flexibility and mobility	Child's pose with lateral reach	30 s per side (1 min total)	Reduction of trunk muscle tension and promotion of trunk mobility
	Relaxation and recovery	Conscious whole-body relaxation	1 min	Reduction of residual muscle tension and completion of the therapeutic exercise session

Week 8 – POSH-Ex Exercise Programme

Training Component	Functional Component	Exercise	Dosage	Primary Objective
**Warm-up (5 min)**	Dynamic warm-up	Marching in place with coordinated arm movements	2 min	Gradual whole-body activation and preparation for therapeutic exercise
	Breathing regulation	Supine diaphragmatic breathing	2 min	Establishment of a controlled diaphragmatic breathing pattern and preparation for coordination of breathing with movement
	Pelvic floor awareness	PFM relaxation coordinated with exhalation	1 min (~10 breathing cycles)	Development of conscious PFM relaxation and proprioceptive awareness in preparation for selective activation
**Main therapeutic exercise component (50 min)**	**1. Neuromuscular activation**	Rapid selective PFM contractions and relaxations in a partial-squat position	3 × 12 repetitions; 1 s contraction / 1 s relaxation; 30 s rest between sets	Progression of rapid selective PFM activation and complete relaxation in a more demanding weight-bearing position
	**2. Lumbopelvic stabilization**	Single-leg bridge with prolonged eccentric lowering	2 × 6 repetitions per leg; 2 s lifting phase; 8 s hold; 4 s lowering phase; 45 s rest between sets	Progression of unilateral lumbopelvic stability and pelvic control through prolonged eccentric lowering
	**3. Postural control**	Single-leg hip hinge with a 2-kg dumbbell held bilaterally	2 × 6 repetitions per side; 4 s lowering phase; 3 s hold; 2 s return phase; 45 s rest between sets	Progression of unilateral postural control, balance, and lumbopelvic stability through slower eccentric movement with external load
		Split-stance trunk rotation with a 2-kg dumbbell	2 × 8 repetitions per side; 3 s rotation; 2 s hold; 3 s return phase; 45 s rest between sets	Development of postural control and thoracic rotation while maintaining pelvic stability, with coordinated PFM activation
	**4. Functional integration**	Squat with a 2-kg dumbbell held in front of the chest	3 × 8 repetitions; 4 s lowering phase; 3 s hold; 2 s return phase; 45 s rest between sets	Progression of loaded functional squat control through slower eccentric loading and prolonged stabilization, with coordinated PFM activation
		Reverse lunge with sustained hold	2 × 8 repetitions per leg; 4 s lowering phase; 3 s hold; 2 s return phase; 45 s rest between sets	Integration of PFM activation and unilateral lower-limb control into a reverse-lunge pattern with increased time under tension
		Lateral lunge with a 2-kg dumbbell	2 × 8 repetitions per side; 4 s lowering phase; 3 s hold; 2 s return phase; 45 s rest between sets	Progression of frontal-plane pelvic and lower-limb control under external load with integrated PFM activation
**Cool-down (5 min)**	Breathing regulation and recovery	Supine diaphragmatic breathing	1 min	Gradual reduction in activity intensity and promotion of relaxation
	Flexibility	Supine hip flexor stretch at the edge of the support surface	30 s per side (1 min total)	Reduction of hip flexor muscle tension and maintenance of hip flexor flexibility
	Flexibility	Quadruped adductor rock-back stretch	30 s per side (1 min total)	Reduction of hip adductor muscle tension and maintenance of hip adductor flexibility
	Trunk flexibility and mobility	Child's pose with lateral reach	30 s per side (1 min total)	Reduction of trunk muscle tension and promotion of trunk mobility
	Relaxation and recovery	Conscious whole-body relaxation	1 min	Reduction of residual muscle tension and completion of the therapeutic exercise session

Week 9 – POSH-Ex Exercise Programme

Training Component	Functional Component	Exercise	Dosage	Primary Objective
**Warm-up (5 min)**	Dynamic warm-up	Marching in place with coordinated arm movements	2 min	Gradual whole-body activation and preparation for therapeutic exercise
	Breathing regulation	Supine diaphragmatic breathing	2 min	Establishment of a controlled diaphragmatic breathing pattern and preparation for coordination of breathing with movement
	Pelvic floor awareness	PFM relaxation coordinated with exhalation	1 min (~10 breathing cycles)	Development of conscious PFM relaxation and proprioceptive awareness in preparation for selective activation
**Main therapeutic exercise component (50 min)**	**1. Neuromuscular activation**	Rapid selective PFM contractions and relaxations in a partial-squat position with lateral weight transfer	3 × 14 repetitions (7 per side); 1 s contraction followed by complete relaxation during weight transfer; 30 s rest between sets	Progression of rapid selective PFM activation and relaxation during dynamic weight transfer in a weight-bearing position
	**2. Lumbopelvic stabilization**	Single-leg bridge with alternating overhead arm movement	2 × 6 repetitions per leg; 2 s lifting phase; 10 s hold with alternating arm movements; 4 s lowering phase; 45 s rest between sets	Progression of unilateral lumbopelvic stability and pelvic control during concurrent upper-limb movement
	**3. Postural control**	Single-leg hip hinge with a 2-kg dumbbell held bilaterally	2 × 6 repetitions per side; 4 s lowering phase; 3 s hold; 2 s return phase; 45 s rest between sets	Maintenance of unilateral postural control, balance, and lumbopelvic stability under external load
		Split-stance trunk rotation with a 2-kg dumbbell	2 × 8 repetitions per side; 3 s rotation; 4 s hold; 3 s return phase; 45 s rest between sets	Progression of postural control and thoracic rotation while maintaining pelvic stability, with coordinated PFM activation
	**4. Functional integration**	Squat with a 2-kg dumbbell and overhead arm elevation	3 × 8 repetitions; 4 s lowering phase; 3 s hold; 3 s return phase followed by overhead arm elevation; 45 s rest between sets	Integration of PFM activation, loaded squat control, and upper-limb movement within a complex functional movement pattern
		Reverse lunge with trunk rotation	2 × 8 repetitions per leg; 4 s lowering phase; 3 s hold with trunk rotation toward the front leg; 2 s return phase; 45 s rest between sets	Integration of PFM activation, unilateral lower-limb control, and trunk rotation within a multiplanar functional movement pattern
		Lateral lunge with a 2-kg dumbbell	2 × 8 repetitions per side; 4 s lowering phase; 3 s hold; 2 s return phase; 45 s rest between sets	Maintenance of frontal-plane pelvic and lower-limb control under external load with integrated PFM activation
**Cool-down (5 min)**	Breathing regulation and recovery	Supine diaphragmatic breathing	1 min	Gradual reduction in activity intensity and promotion of relaxation
	Flexibility	Supine hip flexor stretch at the edge of the support surface	30 s per side (1 min total)	Reduction of hip flexor muscle tension and maintenance of hip flexor flexibility
	Flexibility	Quadruped adductor rock-back stretch	30 s per side (1 min total)	Reduction of hip adductor muscle tension and maintenance of hip adductor flexibility
	Trunk flexibility and mobility	Child's pose with lateral reach	30 s per side (1 min total)	Reduction of trunk muscle tension and promotion of trunk mobility
	Relaxation and recovery	Conscious whole-body relaxation	1 min	Reduction of residual muscle tension and completion of the therapeutic exercise session

Week 10 – POSH-Ex Exercise Programme

Training Component	Functional Component	Exercise	Dosage	Primary Objective
**Warm-up (5 min)**	Dynamic warm-up	Marching in place with coordinated arm movements	2 min	Gradual whole-body activation and preparation for therapeutic exercise
	Breathing regulation	Supine diaphragmatic breathing	2 min	Establishment of a controlled diaphragmatic breathing pattern and preparation for coordination of breathing with movement
	Pelvic floor awareness	PFM relaxation coordinated with exhalation	1 min (~10 breathing cycles)	Development of conscious PFM relaxation and proprioceptive awareness in preparation for selective activation
**Main therapeutic exercise component (50 min)**	**1. Neuromuscular activation**	Rapid selective PFM contractions and relaxations in a partial-squat position with lateral weight transfer	3 × 14 repetitions (7 per side); 1 s contraction followed by complete relaxation during weight transfer; 30 s rest between sets	Maintenance of rapid selective PFM activation and relaxation during dynamic weight transfer in a weight-bearing position
	**2. Lumbopelvic stabilization**	Modified forearm plank with alternating arm elevation	2 × 8 repetitions per arm; 2 s hold; 30 s rest between sets	Development of lumbopelvic stability during unilateral upper-limb movement while maintaining pelvic alignment
		Modified forearm plank with anterior–posterior weight shift	2 × 10 repetitions; 2 s forward shift / 2 s backward shift; 30 s rest between sets	Development of dynamic trunk and lumbopelvic stability during controlled changes in body position and load distribution
	**3. Postural control**	Single-leg hip hinge with a 2-kg dumbbell performed in a continuous rhythm	2 × 8 repetitions per side; 3 s hold; 45 s rest between sets	Progression of unilateral postural control, balance, and lumbopelvic stability during repeated continuous movement under external load
	**4. Functional integration**	Squat with trunk rotation and a 2-kg dumbbell elevation to shoulder height	3 × 10 total repetitions (5 rotations per side); 3 s lowering phase; 2 s hold; 2 s return phase with alternating trunk rotation; 30 s rest between sets	Integration of loaded squat control, trunk rotation, and coordinated PFM activation within a multiplanar functional movement pattern
		Forward lunge with a 2-kg dumbbell lift	2 × 8 repetitions per leg; 3 s hold; 45 s rest between sets	Integration of unilateral lower-limb control, upper-limb loading, and coordinated PFM activation within a functional lunge pattern
		Squat-to-alternating-lunge sequence	2 × 6 per side; 45 s rest between sets	Integration of PFM activation, lower-limb control, coordination, and transitions between consecutive functional movement patterns
**Cool-down (5 min)**	Breathing regulation and recovery	Supine diaphragmatic breathing	1 min	Gradual reduction in activity intensity and promotion of relaxation
	Flexibility	Half-kneeling hip flexor stretch with posterior pelvic tilt	30 s per side (1 min total)	Reduction of hip flexor muscle tension and maintenance of hip flexor flexibility
	Flexibility	Seated butterfly adductor stretch	1 min	Reduction of hip adductor muscle tension and maintenance of hip adductor flexibility
	Trunk flexibility and mobility	Supine trunk rotation stretch	30 s per side (1 min total)	Reduction of trunk muscle tension and promotion of trunk mobility
	Relaxation and recovery	Conscious whole-body relaxation	1 min	Reduction of residual muscle tension and completion of the therapeutic exercise session

Week 11 – POSH-Ex Exercise Programme

Training Component	Functional Component	Exercise	Dosage	Primary Objective
**Warm-up (5 min)**	Dynamic warm-up	Marching in place with coordinated arm movements	2 min	Gradual whole-body activation and preparation for therapeutic exercise
	Breathing regulation	Supine diaphragmatic breathing	2 min	Establishment of a controlled diaphragmatic breathing pattern and preparation for coordination of breathing with movement
	Pelvic floor awareness	PFM relaxation coordinated with exhalation	1 min (~10 breathing cycles)	Development of conscious PFM relaxation and proprioceptive awareness in preparation for selective activation
**Main therapeutic exercise component (50 min)**	**1. Neuromuscular activation**	Rapid selective PFM contractions and relaxations during multidirectional weight transfer	3 × 12 weight transfers (3 in each direction: forward, backward, left, and right); 1 s PFM contraction with each weight transfer followed by complete relaxation; 30 s rest between sets	Progression of rapid selective PFM activation and relaxation during multidirectional dynamic weight transfer in a weight-bearing position
	**2. Lumbopelvic stabilization**	Modified forearm plank with contralateral arm and leg elevation	2 × 8 repetitions per side; 2 s hold; 30 s rest between sets	Progression of lumbopelvic stability during contralateral upper- and lower-limb movement while maintaining pelvic alignment
		Modified forearm plank with circular body shifts	2 × 8 circles in each direction; 30 s rest between sets	Development of dynamic lumbopelvic stability during multidirectional changes in body position and load distribution
	**3. Postural control**	Single-leg hip hinge with overhead dumbbell transfer between hands	2 × 8 repetitions per side; 2-kg dumbbell; 45 s rest between sets	Progression of unilateral postural control, balance, and lumbopelvic stability during multidirectional upper-limb movement and transfer of external load
	**4. Functional integration**	Squat with trunk rotation and dumbbell elevation to shoulder height	3 × 10 total repetitions (5 rotations per side); 30 s rest between sets	Integration of loaded squat control, trunk rotation, upper-limb movement, and coordinated PFM activation within a multiplanar functional movement pattern.
		Forward lunge with trunk rotation and forward dumbbell press	2 × 8 repetitions per leg; 3 s hold; 45 s rest between sets	Integration of unilateral lower-limb control, trunk rotation, upper-limb loading, and coordinated PFM activation within a complex functional movement pattern
		Curtsy (diagonal reverse) lunge	2 × 8 repetitions per side; 2 s hold; 45 s rest between sets	Development of multiplanar lower-limb and pelvic control with coordinated PFM activation
**Cool-down (5 min)**	Breathing regulation and recovery	Supine diaphragmatic breathing	1 min	Gradual reduction in activity intensity and promotion of relaxation
	Flexibility	Half-kneeling hip flexor stretch with posterior pelvic tilt	30 s per side (1 min total)	Reduction of hip flexor muscle tension and maintenance of hip flexor flexibility
	Flexibility	Seated butterfly adductor stretch	1 min	Reduction of hip adductor muscle tension and maintenance of hip adductor flexibility
	Trunk flexibility and mobility	Supine trunk rotation stretch	30 s per side (1 min total)	Reduction of trunk muscle tension and promotion of trunk mobility
	Relaxation and recovery	Conscious whole-body relaxation	1 min	Reduction of residual muscle tension and completion of the therapeutic exercise session

Week 12 – POSH-Ex Exercise Programme

Training Component	Functional Component	Exercise	Dosage	Primary Objective
**Warm-up (5 min)**	Dynamic warm-up	Marching in place with coordinated arm movements	2 min	Gradual whole-body activation and preparation for therapeutic exercise
	Breathing regulation	Supine diaphragmatic breathing	2 min	Establishment of a controlled diaphragmatic breathing pattern and preparation for coordination of breathing with movement
	Pelvic floor awareness	PFM relaxation coordinated with exhalation	1 min (~6 breathing cycles)	Development of conscious PFM relaxation and proprioceptive awareness in preparation for selective activation
**Main therapeutic exercise component (50 min)**	**1. Neuromuscular activation**	Rapid selective PFM contractions and relaxations during multidirectional weight transfer	3 × 12 weight transfers (3 in each direction: forward, backward, left, and right); 1 s PFM contraction with each weight transfer followed by complete relaxation; 30 s rest between sets	Maintenance of rapid selective PFM activation and relaxation during multidirectional dynamic weight transfer in a weight-bearing position
**Main therapeutic exercise component (50 min)**	**2. Lumbopelvic stabilization**	Modified forearm plank with contralateral arm and leg elevation	2 × 8 repetitions per side; 2 s hold; 30 s rest between sets	Maintenance of lumbopelvic stability during contralateral upper- and lower-limb movement while maintaining pelvic alignment
	**2. Lumbopelvic stabilization**	Modified forearm plank with combined multidirectional weight shifts	2 × 10 combined repetitions; approximately 2 s in each direction; 30 s rest between sets	Integration of dynamic lumbopelvic stability during controlled anterior–posterior and lateral changes in load distribution
**Main therapeutic exercise component (50 min)**	**3. Postural control**	Single-leg hip hinge with contralateral diagonal reach using a 2-kg dumbbell	2 × 8 repetitions per side; 3 s hold; 45 s rest between sets	Integration of unilateral balance, lumbopelvic stability, and multiplanar upper-limb movement under external load
**Main therapeutic exercise component (50 min)**	**4. Functional integration**	Squat with trunk rotation and dumbbell elevation to shoulder height	3 × 10 total repetitions (5 rotations per side); 2-kg dumbbell; 30 s rest between sets	Integration of loaded squat control, trunk rotation, upper-limb movement, and coordinated PFM activation within a multiplanar functional movement pattern
	**4. Functional integration**	Forward lunge with trunk rotation transitioning to single-leg balance	2 × 8 repetitions per leg; 2 s hold following trunk rotation; 2 s single-leg balance on the front leg; 45 s rest between sets	Integration of unilateral lower-limb control, trunk rotation, PFM activation, and postural stability during a transitional functional task
	**4. Functional integration**	Reverse lunge to knee drive with trunk rotation using a 2-kg dumbbell	2 × 8 repetitions per side; 2 s hold in the reverse-lunge position; 45 s rest between sets	Integration of PFM activation, dynamic balance, trunk rotation, and lower-limb control during a complex transitional movement
**Cool-down (5 min)**	Breathing regulation and recovery	Supine diaphragmatic breathing	1 min	Gradual reduction in activity intensity and promotion of relaxation
	Flexibility	Half-kneeling hip flexor stretch with posterior pelvic tilt	30 s per side (1 min total)	Reduction of hip flexor muscle tension and maintenance of hip flexor flexibility
	Flexibility	Seated butterfly adductor stretch	1 min	Reduction of hip adductor muscle tension and maintenance of hip adductor flexibility
	Trunk flexibility and mobility	Supine trunk rotation stretch	30 s per side (1 min total)	Reduction of trunk muscle tension and promotion of trunk mobility
	Relaxation and recovery	Conscious whole-body relaxation	1 min	Reduction of residual muscle tension and completion of the therapeutic exercise session
